# Comparison of Many-Particle Representations for Selected Configuration Interaction: II. Numerical Benchmark Calculations

**DOI:** 10.1021/acs.jctc.1c00081

**Published:** 2021-04-22

**Authors:** Vijay Gopal Chilkuri, Frank Neese

**Affiliations:** Max-Planck-Institut für Kohlenforschung, Kaiser-Wilhelm-Platz 1, Mülheim an der Ruhr 45470, Germany

## Abstract

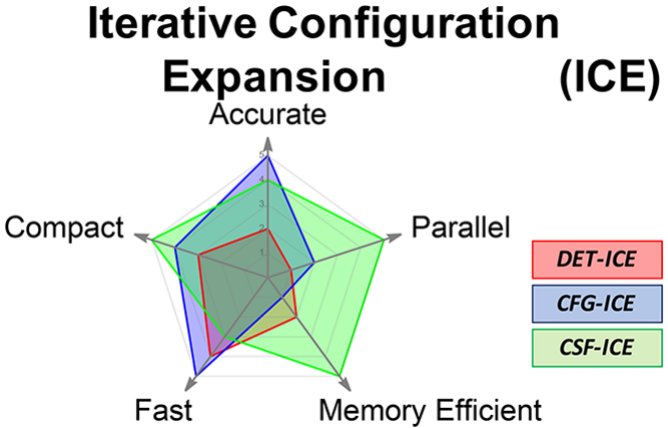

The present work is the second part in our three-part series on the comparison of many-particle representations for the selected configuration interaction (CI) method. In this work, we present benchmark calculations based on our selected CI program called the iterative configuration expansion (ICE) that is inspired by the CIPSI method of Malrieu and co-workers (MalrieuJ. Chem. Phys.1973, 58, ( (12), ), 5745−5759). We describe the main parameters that enter in this algorithm and perform benchmark calculations on a set of 21 small molecules and compare ground state energies with full configuration interaction (FCI) results (FCI21 test set). The focus is the comparison of the performance of three different types of many-particle basis functions (MPBFs): (1) individual Slater determinants (DETS), (2) individual spin-adapted configuration state functions (CSFs), and (3) all CSFs of a given total spin that can be generated from spatial configurations (CFGs). An analysis of the cost of the calculation in terms of the number of wavefunction parameters and the energy error is evaluated for the DET-, CFG-, and CSF-based ICE. The main differences for the three many-particle basis representations show up in the number of wavefunction parameters and the rate of convergence toward the FCI limit with the thresholds of the ICE. Next, we analyze the best way to extrapolate the ICE energies toward the FCI results as a function of the thresholds. The efficiency of the extrapolation is investigated relative to the FCI21 test set as well as near FCI calculations on three moderately sized hydrocarbon molecules CH_4_, C_2_H_4_, and C_4_H_6_. Finally, we comment on the size-inconsistency error for the three many-particle representations and compare it with the error in the total energy. The implication for selected CI implementations with any of the three many-particle representations is discussed.

## 1 Introduction

The selected configuration interaction (sCI) method has recently seen a resurgence and has established itself as a powerful tool for quantum chemistry as evidenced by various recent studies.^1−11^ As the use of sCI methods becomes more widespread, the need for a thorough understanding of various characteristics of sCI methods such as convergence thresholds, extrapolation techniques, and error bars, becomes increasingly important. The absence of comparable experimental data on the one hand and infeasibility of full configuration interaction (FCI) calculations for large molecules on the other renders a rigorous benchmarking of sCI calculations on realistic molecules a rather difficult endeavor. There have been attempts toward a thorough benchmark of sCI methods by various groups recently, such as the benchmarking of the Gaussian-2 set using semistochastic heat-bath configuration interaction^12^ (SHCI) by Yao et al.^13^ Stochastic methods such as the full configuration interaction quantum Monte Carlo (FCIQMC) method pioneered by Alavi and co-workers also have their own standardized algorithms for benchmarking and extrapolation, which depends on the type of FCIQMC algorithm used.^14−17^ Another such effort is illustrated by the recent work of the adaptive sampling CI by Tubman et al.^16,18^ Benchmarking efforts have also been make for the calculation of vertical excitation energies employing sCI methods by Loos et al.^19^ Along with benchmarking efforts, recent collaborative initiatives on comparing various approaches together with sCI have also appeared.^20,21^

A subject that, to the best of our knowledge, has hardly ever been studied before, is the question of which many particle basis is best suited for sCI calculations? Here, we address this question by exploring three different types of many-particle basis functions (MPBFs). The most straightforward choice is to expand the many particle wavefunction in terms of individual Slater determinants (DETs). Alternatively, one can construct individual spin-adapted configuration state functions (CSFs). Lastly, selection can be performed on individual spatial configurations (CFGs) that are then allowed to bring in all CSFs arising from any given configuration. Most existing sCI methods are based on the DET basis and use the total DET count (*N_d_*) as the ordinate for convergence and parallel scaling analysis.^20−23^ These criteria make transferability of thresholds difficult for sCI methods that are based on CFGs and CSFs. In the present work, we examine a general approach for the analysis of the characteristics of a sCI method by comparing the thresholds, extrapolation techniques, and error bars for three different types of MPBFs including individual DETs, CFGs, and CSFs. The similarities and differences between the three MPBFs are presented and discussed. We present the advantages and drawbacks of each type of MPBF in terms of the types of problems adapted for each case.

As a by-product of this work, a systematic benchmark set of 21 small molecules for approximate FCI methods (FCI21) is devised to systematize future benchmarking and comparisons such as those that exist for density functional theory methods.^24,25^ We further augment the FCI21 set with a clear and simple protocol that can be followed to obtain reproducible results for a comparison with other sCI methods and with various types of MPBFs.^26^

This paper is the second in a series of papers on our sCI method called the “iterative configuration interaction” (ICE) method. In Part I of this series, we have presented an in-depth description of the algorithm and implementation details of the ICE in terms of tree data structures and recursive matrix element algorithms.^26^ In the present paper (Part II), we shall describe the numerical performance of the associated thresholds and investigate a simple but effective extrapolation schemes. In Part III, we will present systematic case studies on inorganic and organic molecules for accessing the strengths and weaknesses of the three MPBFs and explore the limits of the ICE method.

The outline of the paper is as follows: First, we briefly recall the algorithm of the ICE method followed by a description of the thresholds entering the protocol. We also describe the methodology for obtaining the statistics from benchmark data. This is followed by the results, which consist of three parts: First, we present the benchmark results on the FCI21 set for the three types of MPBF. Second, we present a general extrapolation scheme for obtaining near FCI energies, and finally, we test the size-inconsistency error as a function of the thresholds of the ICE method. In the conclusions, the main strategies and guidelines for performing calculations using the ICE are outlined.

## 2 Methodology

The details of the algorithm and the associated implementations have been explained in Part I of this series. Therefore, we will only sketch the main steps of the algorithm and provide details about the parts necessary for the benchmarking and extrapolation.

### 2.1 Iterative Configuration Expansion

The ICE algorithm is inspired by the groundbreaking and original configuration interaction by perturbation with multiconfigurational zeroth-order wavefunction selected by iterative process (CIPSI) paper by Huron, Malrieu, and Rancurel,^27^ which appeared in 1973. The original CIPSI algorithm was later modified to a “three class” CIPSI algorithm by Evangelisti et al.^28^ Our ICE algorithm closely resembles the three class CIPSI but differs in some important aspects. The main difference of the ICE with the three class CIPSI algorithm is that the ICE is designed as an approximate full CI method rather than as a multi-reference perturbation theory (MRPT) method. Consequently, emphasis is placed on convergence of the variational energy where possible. Only the selection part relies on MRPT. However, the perturbative energy calculated can be used as a measure of the quality of the initial references during the iterations and for extrapolating to the FCI limit as will be explored below. Second, the ability to work with three different types of MPBFs is a significant difference from both algorithms and has important implications for practical applications. However, the fundamental intellectual basis of this work unambiguously is the pioneering work for Malrieu and co-workers.

**Figure 1:**
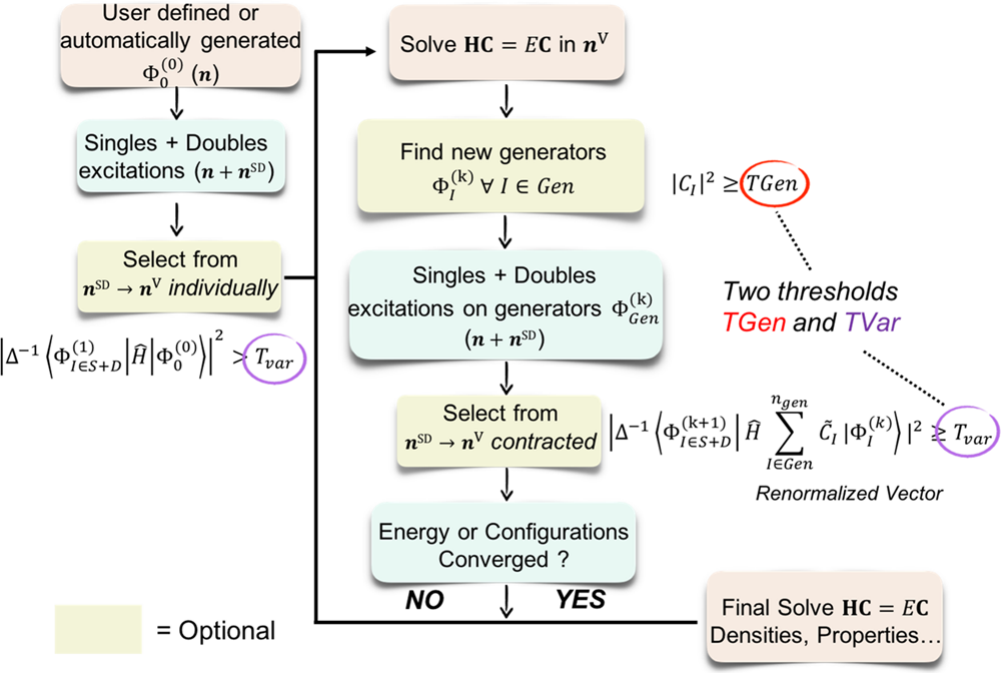
Flowchart of the ICE algorithm. The two parameters that determine the accuracy and convergence criteria are the generator threshold TGen and the variational selection threshold TVar.

The algorithm is summarized in Figure 1.

The main steps of the algorithm which has been implemented in the ORCA program package^29−31^ are as follows:

a) The ICE procedure is “seeded” by a rationally chosen |0⟩^th^ order set of MPBFs |Φ_*I*_^(0)^⟩ ∈|Ψ_0_⟩, which are expected to represent a dominant part of the state of interest. However, this is not strictly required as the algorithm will also find states that have not been anticipated. Other than a manually input set of initial MPBFs, |Ψ_0_⟩ can be also be constructed automatically by performing an initial complete active space self-consistent field (CASSCF) calculation with a smaller CAS space. Here, the |Φ_*I*_^(0)^⟩′*s* ∈ Ψ*_CAS_* will correspond to the CASSCF root of interest.
b) An initial selection is performed by generating the possible single and double excitations relative to the initial configurations. The selection is performed by evaluating the second-order Epstein–Nesbet perturbation energy^32,33^ (PT2) relative to all initial MPBFs individually. Excited MPBFs with a perturbation contribution of larger than the first threshold TVar are included in the (“selected”) variational space.
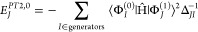
1where the denominator Δ_*JI*_^–1^ is the energy difference and is given by eq 2 below.
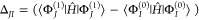
2
c) The many-particle Hamiltonian is diagonalized over the set of the set of presently selected MPBFs, thus defining the initial many particle states.
d) The eigenfunctions arising from the diagonalization are analyzed with respect to the leading contributions to the roots found. MPBFs with a weight of larger than the second threshold TGen are considered as “generator” MPBFs. This is also illustrated in the flowchart of the ICE algorithm in Figure 1.
e) The generator part of the present wavefunction is renormalized thus defining the “contracted generator wavefunction”. The renormalized wavefunction and energy are obtained by diagonalizing the Hamiltonian in the generator space as given by eqs 3–6 below. The reason being that the PT2 expressions given in eqs 1 and 6 are valid only if the zeroth-order wavefunction (∑_*I* ∈ generators_*C_I_*|Φ_*I*_^(0)^⟩) is an eigenfunction of the Hamiltonian.^34,35^
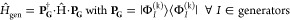
3
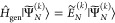
4
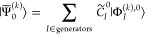
5Here, **P_G_** is the projector in the generator space and *Ĥ*_gen_ is the projected Hamiltonian. Care has to be taken to ensure that the energetic ordering of the states is maintained in the projected Hamiltonian *Ĥ*_gen_.
f) For the subsequent selection that generates all single and double excitations from each of the generator MPBFs. Here, the selection is performed with respect to the interaction of the excited MPBFs and the contracted generator wavefunction and MPBFs with perturbation energies larger than TVar are added to the variational space.
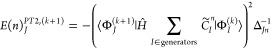
6where Δ_*Jn*_^–1^ represents the energy denominator for the contracted selection for the *n*th root and is given by eq 7
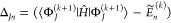
7Here, the quantities *C̃*_I_^n^ and *Ẽ*_n_^(*k*)^ are the renormalized coefficients and energy at the *k*th iteration for the *n*th root, respectively.
g) Convergence of the wavefunction and energy is checked. If convergence has not yet been achieved, the algorithm proceeds by going back to step (d). The energy change from *k* to *k* + 1 iteration can be calculated as Δ*E_n_* = *E*_n_^k^ – *E*_n_^*k* + 1^. Once the energy difference at the *k* + 1th level is smaller than a small, predefined value (e.g., 10^–14^), the calculation is considered to be converged.
h) For a multi-state ICE, we follow the strategy of state-averaging. Hence, before selection begins, the PT2 contribution of each new MPBF needs to be summed for all roots as shown in eq 8 below. Once the PT2 energy *E*_J_^*PT*2, *k* + 1^ for all the newly generated MPBFs has been calculated, the selection of important new MPBFs can be made. Here again, TVar dictates the value of *E*_J_^*PT*2, *k* + 1^ beyond which a newly generated MPBF is to be included in the variational space, i.e., *E*_J_^*PT*2, *k* + 1^≥ TVar. Note that a vast majority of the generated MPBFs will be rejected and only a small number will satisfy the criteria listed before as will be shown later. Importantly, the total PT2 energy *E*_rest_^*PT*2, (*k* + 1)^ of those MPBFs that have been rejected can then be estimated by summing over their contribution over all states.

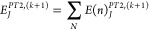
8

The rest of the steps are similar to the single-state ICE.

The three parameters that will be required in the following benchmarking analysis are the two thresholds TGen and TVar and PT2 energy of the discarded MPBFs *E*^*PT*2^ = ∑*_J_*E_J_^*PT*2^, also referred to as the “rest” energy, which will be used during the extrapolation.

In the following analysis, we shall use the combined parameter , which is convenient for compressing TGen and TVar into one single parameter that controls the overall accuracy (and cost) of the algorithm. Where it becomes necessary to show both TGen and TVar, e.g., in the extrapolation section, it is convenient to label the calculation with the two parameters TGen and τ as ICE(*A*, τ) (*A* = – Log_10_(TGen)). For example, a calculation with TGen =10^–4^ and TVar =10^–11^ will be labeled as ICE(4,7).

### 2.2 Statistics and Error Estimation

The natural reference for the ICE methods is provided by actual FCI calculations. Hence, the FCI21 benchmark set is chosen such that FCI calculations are possible on all the molecules included. Benchmarking mainly concerns the study of the error in the ICE energy *E_ICE_* vs the FCI energy *E_FCI_* as a function of the two thresholds TGen and TVar. In order to test the largest spread of the values of TGen and TVar, we performed calculations in steps of factors of 10. The range for TGen is chosen to be from 10^–2^ to 10^–8^ and for TVar from 10^–6^ to 10^–14^, thus leading to a total of 64 calculations for each of the 21 molecules and a total of 1344 data points. The average error was estimated by taking the mean of the 21 molecules for each pair of TGen and TVar values. Note that since the ICE is a variational method, the errors in the energy with respect to FCI are always positive. The error bars were estimated by calculating the variance σ^2^ of the error in the 21 molecules for each pair of TGen and TVar parameters.^34,35^

## 3 Benchmark Results

The accuracy of the ICE algorithm for DETs, CFGs, and CSFs is performed by comparison of the molecules in the FCI21 set with the FCI energy. The cc-pVDZ double-ζ basis set^36^ was used for all the molecules except N_2_, O_2_, F_2_, and CH_4_ for which the Ahlrichs split valence (SV)^37^ basis (without polarization functions) was kept on the heavy atoms in order to make the FCI calculations feasible. The geometry was optimized at the FCI level for the ground state with the given basis set, the converged distances, and angles are given in Table 1, which also shows the number of electrons and orbitals correlated for each molecule. The coupled-cluster energies for closed shell molecules was also calculated at CCSD, CCSD(T), CCSDT, and CCSDT(Q) levels of theory using the MRCC program of Kállay et al.^38^ The converged FCI energies along with the coupled-cluster energies for the FCI21 set are given in Table 2 below.

**Table 1:** Geometries and the Dihedral Angles for the 21 Diatomic Molecules Used in the Present Benchmarking Set^a^

molecule	distance (Å)	angle (^°^)	dihedral (^°^)	ground state	FCI dim.
H_2_	0.7609			^1^Σ^+^	(2e,10o)
LiH	1.6136			^1^Σ^+^	(4e,19o)
BeH	1.3570			^2^Σ^–^	(5e,19o)
BH	1.2551			^1^Σ^+^	(6e,19o)
CH	1.1424			^2^Σ^–^	(7e,19o)
NH	1.9863			^1^Σ^+^	(8e,19o)
OH	0.9796			^2^Σ^–^	(9e,19o)
FH	0.9200			^1^Σ^+^	(10e,19o)
Li_2_	2.7139			^1^Σ_g_^+^	(6e,28o)
Be_2_	4.4269			^1^Σ_g_^+^	(8e,28o)
Li_2_	2.7139			^1^Σ_g_^+^	(2e,26o)
Be_2_	4.4269			^1^Σ_g_^+^	(4e,26o)
B_2_	1.6531			^1^Σ_g_^+^	(6e,26o)
C_2_	1.2728			^1^Σ_g_^+^	(8e,28o)
N_2_	1.1368			^1^Σ_g_^+^	(10e,16o)
O_2_	1.2786			^3^Σ_g_^–^	(12e,16o)
F_2_	1.4186			^1^Σ_g_^+^	(14e,16o)
CH_4_	1.1015	109.5	120.0	^1^A_1_	(8e,28o)
			240.0		
NH_3_	1.0277	103.5	107.7	^1^A_1_	(8e,28o)
H_2_O	0.9668	101.9	0.0	^1^A_1_	(8e,23o)
HF	0.9203			^1^Σ^+^	(8e,18o)

a The ground state is given in *D*_∞*h*_, *C*_∞*v*_, or the highest Abelian symmetry of the molecule.

**Table 2:** List of 21 Molecules Used for Benchmarking the ICE TGen and TVar Parameters^a^

molecule	FCI	CCSD	CCSD(T)	CCSDT	CCSDT(Q)
H_2_	–1.163673	–1.163673			
LiH	–8.014803	–8.014792	–8.014792	–8.014803	–8.014803
BeH	–15.189297				
BH	–25.216401	–25.213458	–25.214831	–25.215255	–25.215307
CH	–38.382084				
NH	–55.026422	–55.009514	–55.016383	–55.024254	–55.024839
OH	–75.561655				
FH	–100.230595	–100.226228	–100.228149	–100.228246	–100.228660
Li_2_	–14.901465	–14.901405	–14.901460	–14.901463	–14.901465
Be_2_	–23.235159	–23.234835	–23.235080	–23.235136	–23.235153
Li_2_	–14.900671				
Be_2_	–29.2343				
B_2_	–49.252200	–49.228166	–49.243773	–49.246299	–49.249561
C_2_	–75.730031	–75.697370	–75.726797	–75.726013	–75.731056
N_2_	–109.016590	–109.005099	–109.014143	–109.014165	–109.016627
O_2_	–149.672045				
F_2_	–198.757772	–198.750591	–198.756217	–198.756318	–198.757841
CH_4_	–40.322818	–40.319708	–40.322373	–40.322699	–40.322813
NH_3_	–56.403537	–56.398826	–56.402861	–56.403180	–56.403544
H_2_O	–76.242083	–76.238216	–76.241384	–76.241591	–76.242139
HF	–100.228876	–100.226235	–100.228226	–100.228349	–100.228805

a The FCI and coupled-cluster energies for closed shell molecules is also shown.

### 3.1 Comparison of Variational ICE vs FCI Energy

**Figure 2:**
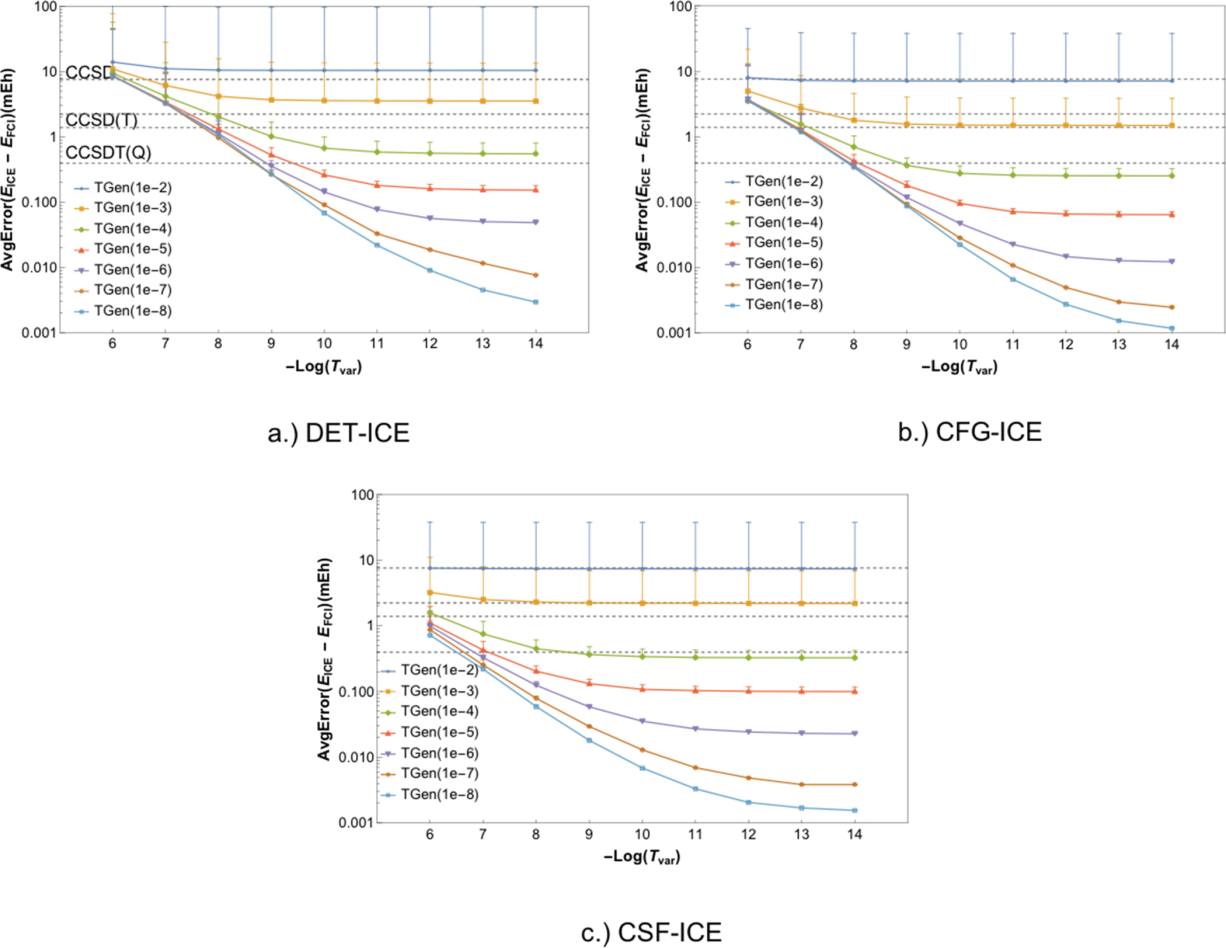
FCI21 benchmark set average error in the variational ICE energy for DET, CFG, and CSF-ICE vs TGen and TVar shown in (a), (b), and (c), respectively. The vertical lines represent the variance in the error for the 21 molecules. TVar is shown on the *x* axis with decreasing values of TGen as shown by the different curves. The coupled-cluster average errors are shown as horizontal lines for comparison.

**Figure 3:**
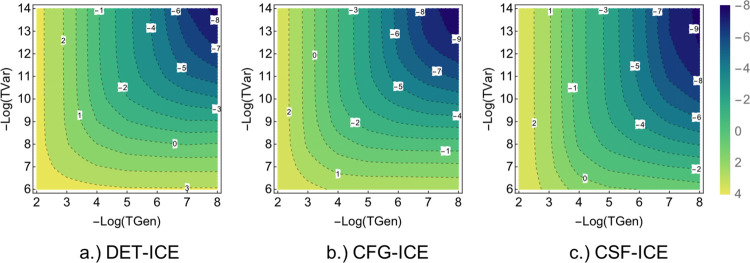
Comparison of the average error as a function of the two thresholds TGen and TVar. The average error for the DET, CFG, and CSF-ICE are shown in Log_2_[Δ*E*] mH.

In order to clearly present all the data and the various aspects of the error analysis with TGen and TVar thresholds, two types of plots are chosen. First, the error vs TVar is plotted in order to show the rate of convergence with the parameters along with the variance in the error for the FCI21 set. In the second type of plot, the variation of the average energy error is shown in a contour plot with TGen and TVar together to facilitate the comparison of DET, CFG, and CSF ICE. In this way, all the different aspects of the dependence of error and error bars with TGen and TVar can be studied. Each point in Figures 2a–c and 3 gives the average of the error together with the variance for each of the 21 molecules as described in Section 2.2.

From inspection of Figure 2, the following observations are made:

a) Decreasing TVar with a fixed value of TGen leads to a plateau behavior, and hence decreasing TVar beyond the plateau does not improve the error. This implies that in order for there to be substantial change in the wavefunction, TGen has to be decreased. Actually, the excitation order of MPBFs in |Ψ*^k^*⟩ (with respect to |Ψ_0_⟩) is controlled by TGen, whereas TVar has no effect on the excitation order. Therefore, for a sufficiently small TVar, an improvement in energy is achieved by adding functions, which have a higher excitation order compared to those present in |Ψ^*k* – 1^⟩. This precisely is what is achieved by decreasing TGen.
b) The convergence with TGen for a given sufficiently small TVar is exponential for all three variants of the ICE. This is very satisfying since this implies that one does not have to tighten both TGen and TVar for approaching the FCI limit. It is enough to fix a sufficiently large TGen/TVar ratio (as done by introducing the parameter τ) and then decrease TGen to systematically approach the FCI energy. This strategy will be used in the extrapolation scheme described below.
c) The plateau behavior with TGen for a fixed large TVar is less pronounced with CFG-ICE and CSF-ICE. However, the DET-ICE variant shows a plateau with TGen for a fixed large TVar as can be seen in Figure 3a.
d) The error bars giving the variance of the energy have a constant value with decreasing TVar for a fixed TGen. Therefore, it appears that the variance decreases mainly with a decrease in TGen and is unaffected by decreasing TVar.
e) The variance in the error of the FCI21 set slightly decreases upon going from DET, CFG to CSF-ICE, as expected. First, this is due to the generally larger absolute error for DET-ICE compared to CFG-ICE and CSF-ICE with a given TGen and TVar. The second reason is due to the normalization of the wavefunction. The CFG and CSF MPBFs are more compact compared to the DET MPBF, and hence the wavefunction expansion in CFG and CSF basis is shorter than that in the DET basis. This implies that for a given threshold TGen and TVar, the wavefunction |Ψ*^k^*⟩ will be closer to the FCI one for the CFG and CSF MPBF than the DET MPBF irrespective of the type of molecule.

A comparison of variations with both TGen and TVar together can be made using a contour plot of the error versus the two parameters as shown in Figure 3a–c for DET, CFG, and CSF-ICE, respectively, below. As one can see, the behavior is qualitatively similar for the DET, CFG, and CSF MPBF, as expected. More interestingly, the convergence with TGen is faster than the convergence with TVar for CFG-ICE and CSF-ICE as can be seen from the steepness of the contours. However, for DET-ICE, the dependence on TGen and TVar are more symmetrical. Next, we shall analyze the convergence of the “rest” PT2 contribution and the effect of adding it to the variational energy.

### 3.2 Comparison of Variational and PT2 ICE vs FCI Energy

In this section, we present a comparison of the variation and perturbative energy for the FCI21 benchmark set. Before presenting the data for the three types of MPBFs, we first present an illustrative example to highlight the difference between DET, CFG, and CSF MPBFs during the calculation of the perturbative energy contributions.

#### 3.2.1 Illustrative Example: Butadiene

**Figure 4:**
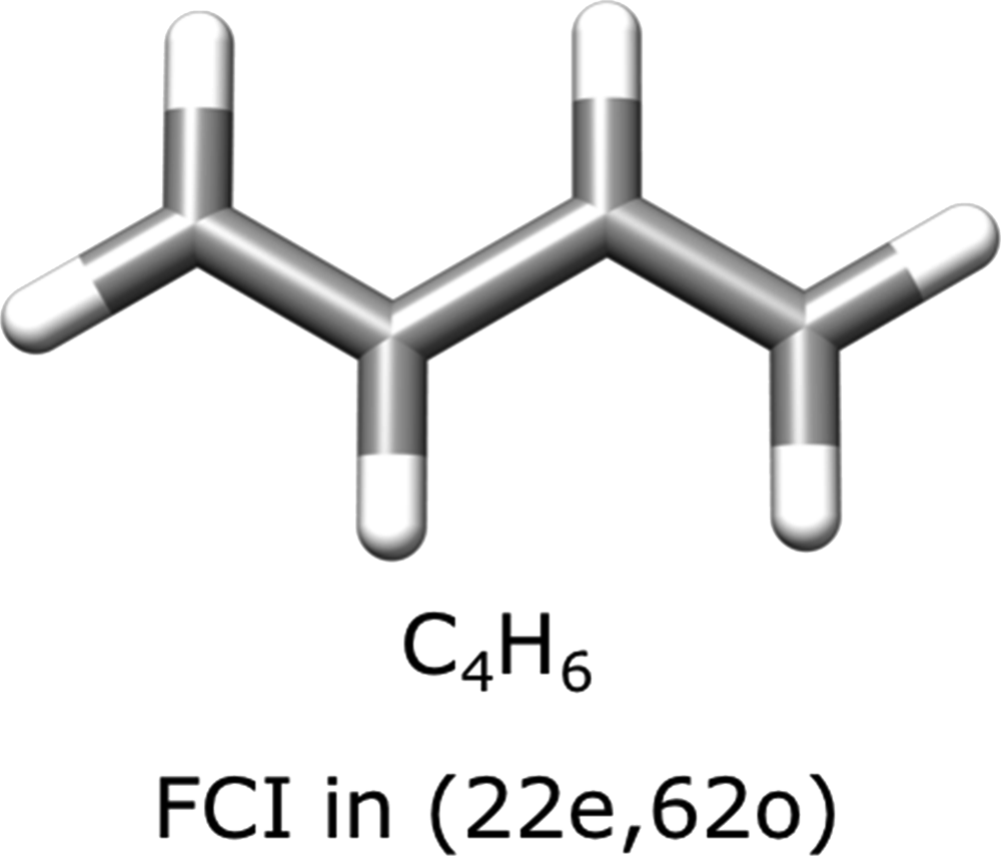
Butadiene molecule used in the analysis of the PT2 energy contribution with DET, CFG, and CSF MPBFs. The cc-pVDZ basis is taken for hydrogen atoms and the SV basis for the carbon atoms resulting in a FCI space of (22e,62o).

The calculation of the perturbative energy estimate of the singly and doubly excited MPBFs from a set of generator MPBFs is achieved using Epstein–Nesbet partitioning of the Hamiltonian as described in Section 2.1. In order to understand the difference in the perturbative energy calculation between the DET, CFG, and CSFs MPBFs, first we shall show a simple example of the butadiene (C_4_H_6_) molecule (see Figure 4 below).

**Figure 5:**
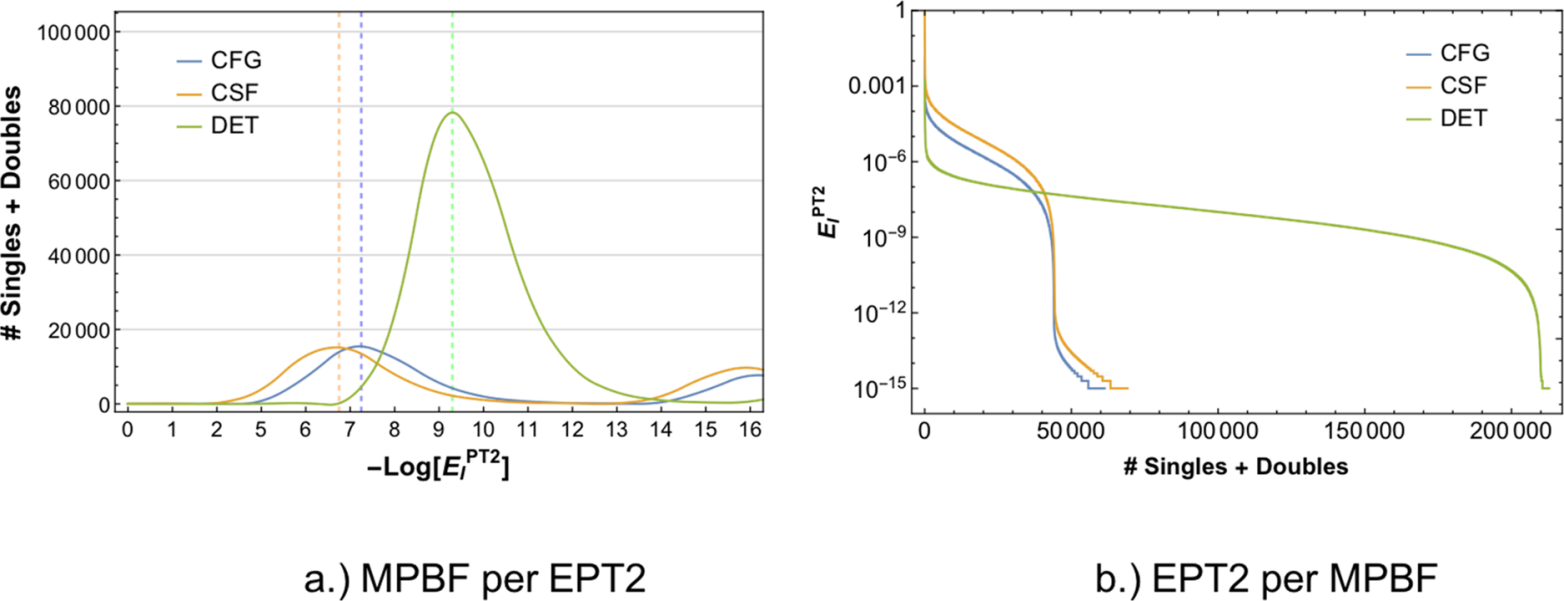
Perturbative energy calculation on the butadiene molecule with a FCI space of (22e,62o) using the cc-pVDZ basis for hydrogen and SV basis for carbon. The RHF configuration is chosen as the |0⟩th order wavefunction |Ψ_0_⟩. a.) The PT2 energy density defined as the number of MPBFs with a given magnitude of *E*_I_^*PT*2^ energy contribution (in millihartree). The distribution is close to an asymmetric Gaussian with long tails, as expected. The individual contribution of each MPBF is given in panel (b). The total number of singles and doubles is larger for DET MPBF than CFG and CSF MPBFs, as expected. The dashed lines indicate the peak values of the PT2 contributions for each curve to enable a quantitative comparison.

The butadiene molecule (cc-pVDZ basis for hydrogen atoms and SV basis for the carbon atoms) has 62 orbitals and a total of 22 electrons (22e,62o). The closed shell restricted Hartree–Fock (RHF) configuration (i.e.,[2 2 2 2 2 2 2 2 2 2 2 0...0] ) is chosen as the |0⟩th order wavefunction |Ψ_0_⟩. The perturbative contribution of all the singly and doubly excited MPBFs starting from |Ψ_0_⟩ in DET, CFG, and CSF MPBFs has been calculated and is given in Figure 5 below.

The butadiene molecule was chosen for a demonstrative purpose here since the space of singly and doubly excited MPBFs from |Ψ_0_⟩ is large enough for a somewhat general comparison of the behavior of perturbative energy contribution in the DET, CFG, and CSF MPBFs. The main conclusions from Figure 5 showing the spread of the perturbative contribution for the three many-particle representations are the following:

a) The CFG and CSF many-particle representations show a similar spread of the perturbative contribution. Both MPBFs contain about a maximum of 50,000 MPBFs having a perturbative energy contribution of *E*_I_^*PT*2^ ≥ 10^–7^ mEh as shown in Figure 5b. Note that the total number of singly and doubly excited MPBFs in the CFG and CSF basis is the same and is 158,202.
b) The DET many-particle representation on the other hand has a total of 912,186 single and doubly excited DETs, which is about 1 order of magnitude larger than the CFG and CSF MPBFs. This is not unexpected as DETs are expanded in an *M_s_* = 0 basis and not a spin eigenbasis.
c) The spread of the perturbative energy contribution for the DET basis is much larger than CFG and CSF basis. As shown in Figure 5b, there are about 200,000 DETs that have a non-negligible perturbative energy contribution compared to about 50,000 for the CFG and CSF case. This is due to the fact that every double excitation on a closed shell configuration generates a single configuration, which can be made up of one, two, four, or six determinants. This indicates that at least four times more DETs (than CSFs) are required for taking into account all MPBFs with a non-negligible perturbative energy contribution at least for C_2_H_4_.
d) A direct consequence of this larger spread in the DET basis is that the perturbative contribution brought in by a single DET is about 1 order of magnitude smaller than that of a single CSF. This is more clearly seen in Figure 5a, which shows the total number of MPBFs that have a given *E*_I_^*PT*2^ contribution.
e) From the comparison of the PT2 energy density for DET, CFG, and CSF MPBFs given in Figure 5a, it is easy to identify the average value of *E*_I_^*PT*2^ brought in by a single MPBF. This value is about 10^–7^ mEh for CFG and CSF MPBF and about 10^–9^ mEh for DET MPBF. Therefore, the energy brought by most of the DETs is more than two orders of magnitude smaller than that for the CFG and CSF MPBFs.
f) This average value of *E*_I_^*PT*2^ can be used in order to estimate the minimum values of the thresholds TGen and TVar adequate for a reliable FCI energy approximation.

#### 3.2.2 Benchmark Results

Now, we shall present the analysis on the FCI21 benchmark data. Once the new MPBFs are chosen as described in Section 2.1, the perturbative energy contribution of the rejected set of MPBFs can be added to the total energy in order to estimate the FCI value as given in eqs 9 and 10 below:
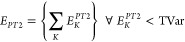
9
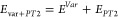
10

The following are two points about the “rest” PT2 energy (*E*_*PT*2_) that need to be emphasized:

a) As the perturbative estimate is calculated using the Epstein–Nesbet zeroth-order Hamiltonian, it will tend to be overestimated.^37,38^ The extent of this overestimation needs to be benchmarked as a function of TGen and TVar. It is expected that the CFG-ICE will show a relatively larger overestimation due to the fact that all the CSFs of a given singly or doubly excited CFG are included while calculating its PT2 contribution.
b) Note that since only the generator MPBFs are used to calculate the perturbative contribution, part of the PT2 correction due to the “spectator” MPBFs is lost and a bias is introduced. On the other hand, this PT2 contribution due to generators comes essentially free of cost, whereas a full PT2 contribution would require additional non-trivial computational effort. This bias can be systematically removed upon decreasing the TGen threshold.

**Figure 6:**
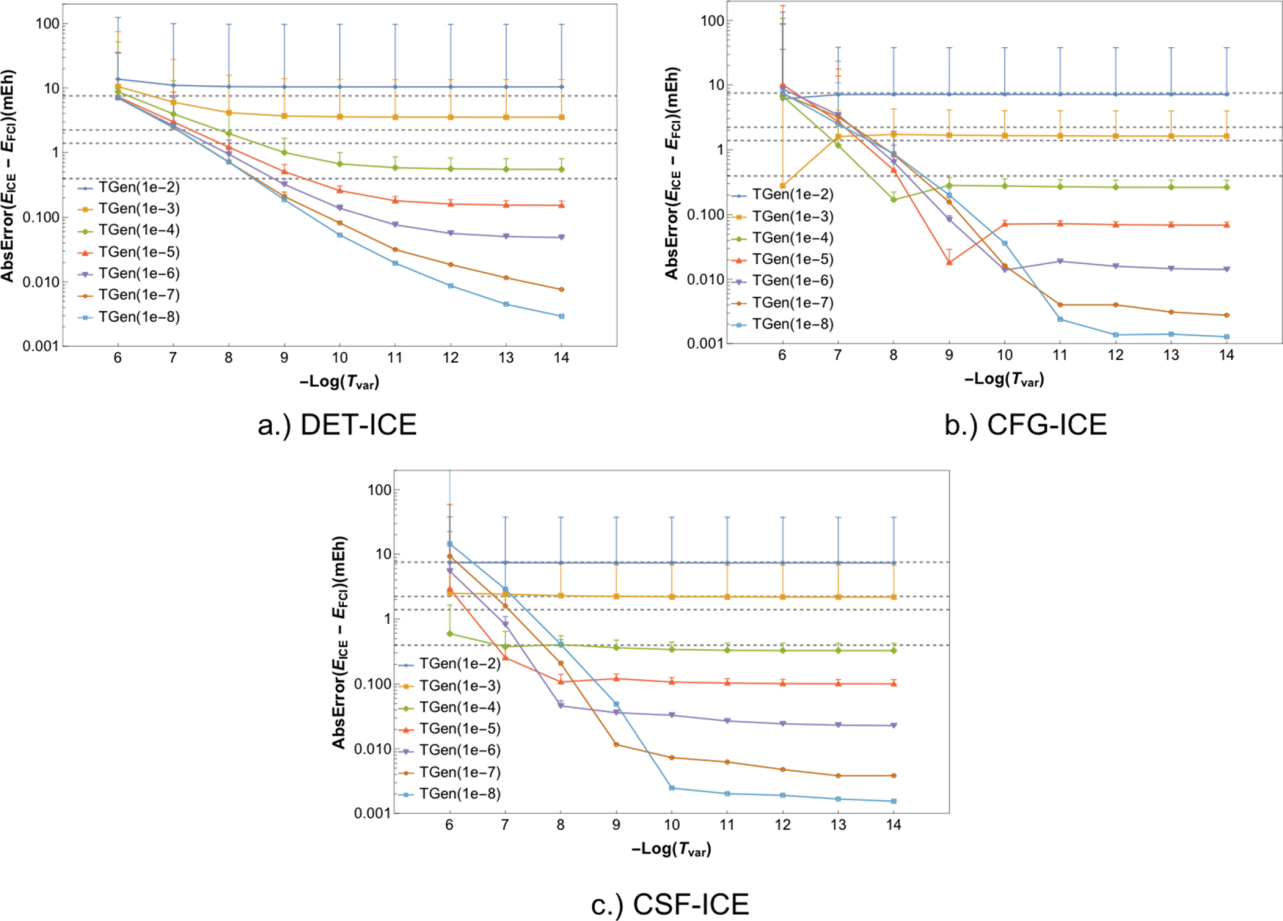
Absolute errors for the DET, CFG, and CSF-ICE including the perturbative correction due to the MPBFs not included in the variational space. Panels (a), (b), and (c) give the absolute error for DET, CFG, and CSF-ICE respectively.

The error in the energies with the “rest” PT2 correction of the FCI21 set can be compared to the FCI results for the three variants as given in Figure 6a–c for DET-ICE, CFG-ICE, and CSF-ICE respectively. Note that in contrast to the variational energy, the PT2 corrected energies can become lower than the FCI energy. In order to plot all values with the thresholds TGen and TVar, the absolute value of the energy error is plotted in the figures below.

There is a range of interesting observations and differences between the DET, CFG and CSF-ICE numbers. The major points are summarized below:

a) The PT2 correction for the DET-ICE behaves very differently compared to the CFG-ICE and CSF-ICE. The main reason for this fundamental difference is due to the fact that the PT2 contribution due to a single DET is in general much smaller than the PT2 contribution due to a single CSF belonging to the same configuration as shown in the previous section. Consequently, the thresholds cannot be directly compared for DET-ICE and CSF/CFG-ICE.
b) The most significant observation is that the perturbative energy estimate is larger by at least an order of magnitude for the CFG-ICE and CSF-ICE compared to the DET-ICE, thus corroborating the finding of the previous section. For the DET-ICE, even with a small TGen value of 10^–6^ and large TVar value of 10^–6^, the perturbative contribution is negligible compared to the variational correlation energy. However, for the CFG-ICE and CSF-ICE, the perturbative contribution for TGen 10^–6^ and TVar 10^–6^ is so large that the total energy error increases from 1 to about 10 mH as can be seen in Figure 6b,c. This also follows from the analysis in the previous section where the average energy brought in by a single CSF was shown to be about 10^–6^ mH.
c) The *E*_*var* + *PT*2_ energy error increases with decreasing TGen ( and a fixed TVar) for the CFG-ICE and CSF-ICE variant due to the fact that upon increasing TGen (i.e., moving down vertically in Figure 6b and Figure 6c), the PT2 contributions become larger. This is because the Epstein–Nesbet PT2 theory tends to overestimate the PT2 contribution and, as a result, the total energy overshoots, becoming more negative compared to the FCI energy. Therefore, the PT2 absolute error increases with decreasing TGen for a fixed TVar.
d) The variance of the errors shows a similar behavior as compared to the variational energies.

**Figure 7:**
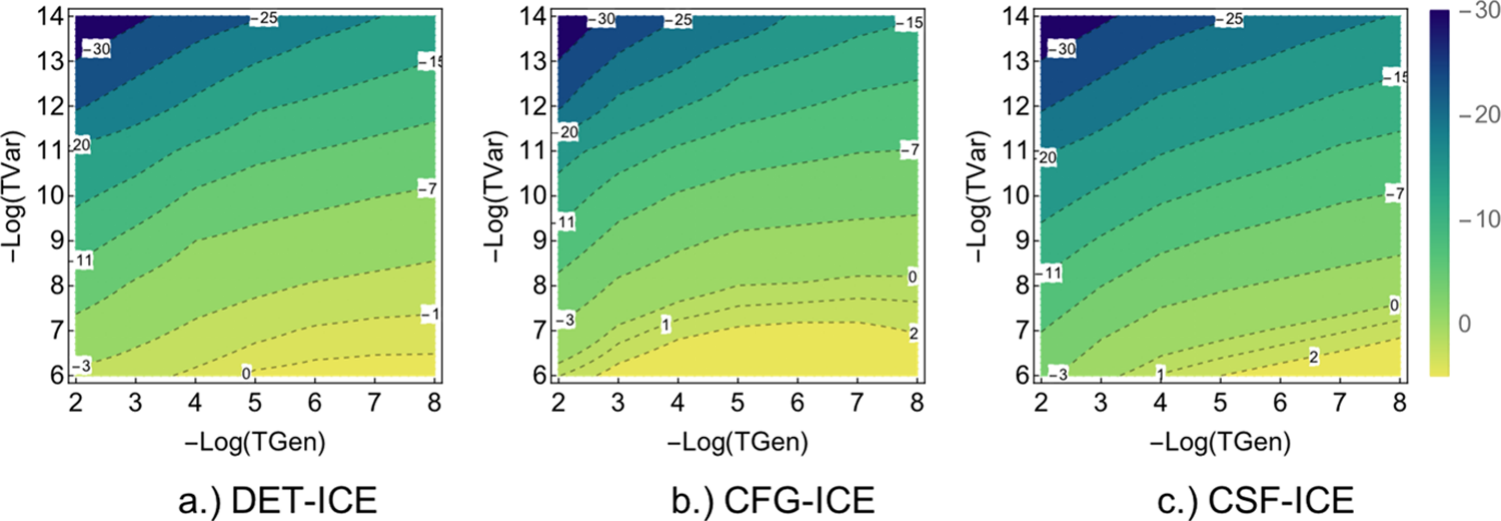
Comparison of the Log of the absolute values of the PT2 contribution Log_2_[|Δ*E*^*PT*2^|] in mEh for the DET, CFG, and CSF MPBF. The bright yellow regions represent a large Δ*E*^*PT*2^, and dark regions represent vanishing Δ*E*^*PT*2^ contributions.

In order to better understand the *E*_*PT*2_ contribution, in Figure 7a–c, we show a contour plot of the log of the absolute value of the perturbative energy *E*_*PT*2_ (i.e., Log_2_[|Δ*E*^*PT*2^|]) for the DET-ICE, CFG-ICE, and CSF-ICE with TGen and TVar thresholds. As expected, there is a large PT2 contribution for larger values of TVar as shown by the horizontal yellow block in Figure 7 for all types of MPBFs. This is due to the fact that, as TGen decreases, the ICE tends to an MRPT type method with an EN zeroth-order Hamiltonian, which is known to overestimate the PT2 contribution.^39,40^ However, the perturbative contribution decreases exponentially with decreasing TVar. Note that, as remarked earlier, the PT2 contribution is largest for the CFG-ICE as shown by the yellow region in Figure 7b.

Therefore, not surprisingly, small values of TGen and TVar seem to be ideal for an accurate prediction of the FCI energy. However, as we shall analyze in detail in the next section, decreasing the thresholds implies an increase in the total number of variational parameters in the wavefunction.

In summary, adding the PT2 energy to the variational ICE energy is a mixed blessing. On one hand, it can reduce the error of the calculation relative to the FCI results. On the other hand, the convergence to the FCI limit is far less smooth due to the overshooting of the Epstein–Nesbet second-order energy and one can also undershoot the FCI energy. Hence, extrapolation appears to be a more promising strategy to improve the variational ICE results as will be discussed below.

### 3.3 Comparison of Variational Parameters

As the solution of a large eigenvalue problem is the rate-limiting step in a sCI procedure, the number of variational parameters in DET, CFG, and CSF representation plays a major role in determining the efficiency of the method. Clearly, the number of variational parameters depends on the values of the two thresholds TGen and TVar in addition to the choice of the MPBF used. As the thresholds approach zero, the number of variational parameters (i.e., number of CSFs or DETs) approaches their respective FCI dimensions, which can be prohibitive. Analysis of the number of variational parameters with respect to the two thresholds TGen and TVar can provide important information about the compactness of the wavefunction for the respective threshold regimes. In the present section, we study the increase in the number of wavefunction parameters for the three variants of the ICE as a function of the two parameters TGen and TVar.

**Figure 8:**
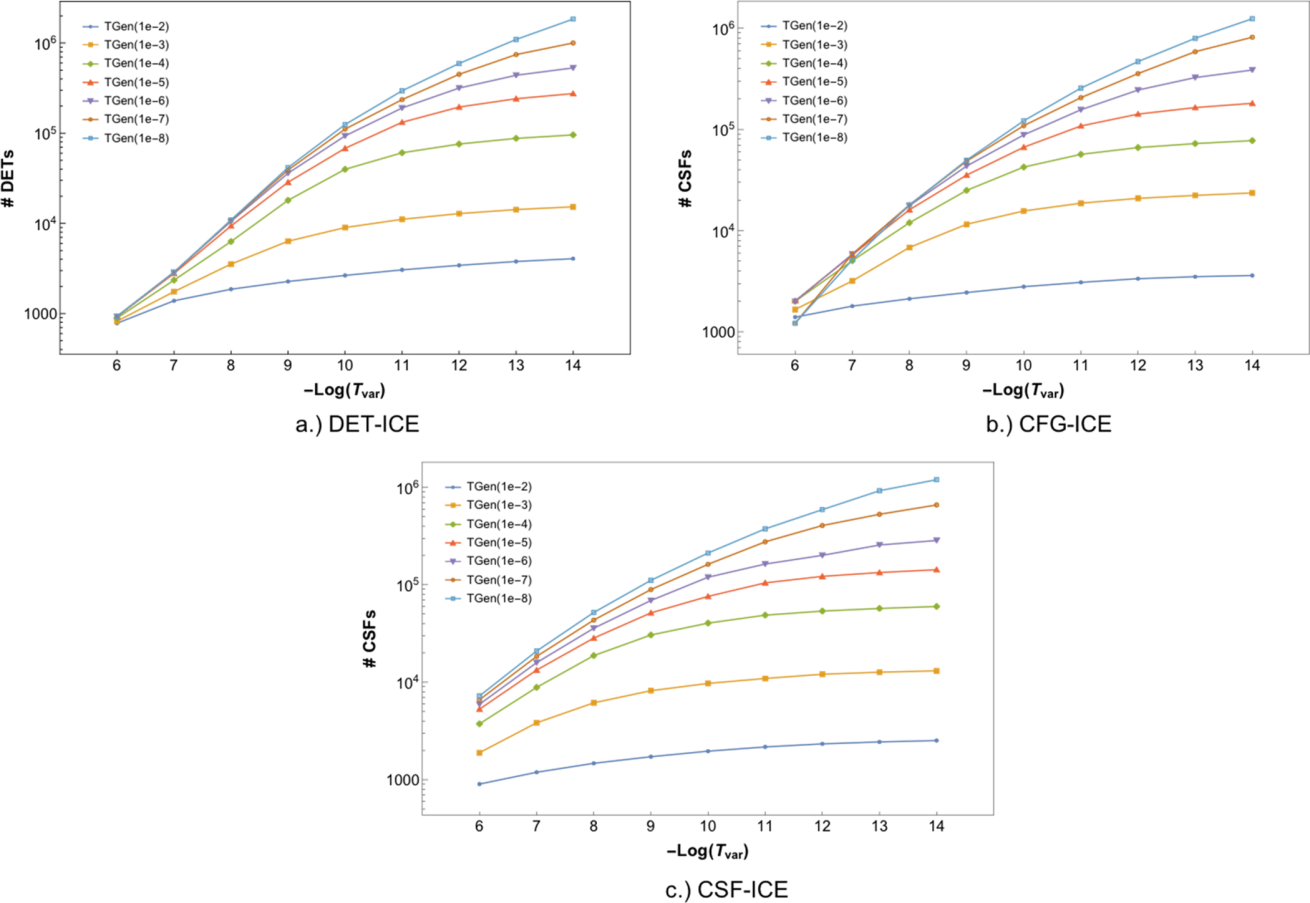
Comparison of the total number of MPBFs in the variational space for the (a) DET-ICE, (b) CFG-ICE, and (c) CSF-ICE variants with TGen and TVar. The data for the plots is taken from the average values corresponding to the FCI21 set.

Figure 8 shows the variation of the wavefunction parameters with the two thresholds TGen and TVar. As expected, the number of variational parameters increases with decreasing thresholds for all three representations. Notice that although the number of wavefunction parameters keeps on increasing with a decrease in TVar (and fixed TGen), the total error in energy stays constant as shown in Figure 2. Therefore, beyond a certain value of TVar, including more MPBFs of the same excitation order (due to fixed TGen) does not improve the wavefunction. This observation is in agreement with the analysis of 3.2.1 where it was shown beyond a threshold (10^–6^ for CSF/CFG and 10^–9^ for DET) adding additional MPBFs has a negligible effect on the total energy.

**Figure 9:**
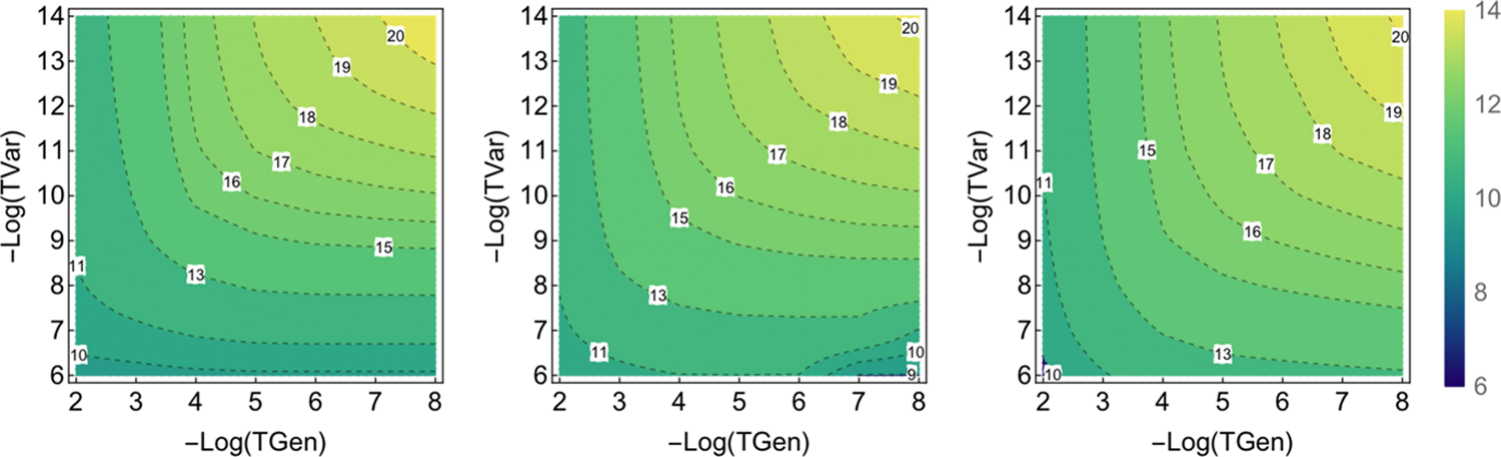
Comparison of the logarithm of the number of wavefunction parameters (DETs or CSFs) (i.e., Log_2_[# MPBFs]) for the three many-particle representations plotted against the two thresholds TGen and TVar.

The number of wavefunction parameters also increases upon a decrease in TGen. A comparison of the logarithm number of wavefunction parameters with both thresholds TGen and TVar together is given in Figure 9 where a contour plot is shown with the number of wavefunction parameters against TGen and TVar. For all three MPBFs, there is a similar rate of increase in the number of parameters with a decrease in TGen or TVar.

The increase in the number of wavefunction parameters with decreasing thresholds is accompanied by a decrease in the error in the energies with respect to the FCI values. Notice that, as one would expect, the smallest energy error occurs at the top right corner of Figure 3, which also corresponds to the largest magnitude of parameters (see Figure 9). There is a direct connection between the number of MPBF and the computational time required for the calculation. In the next section, we shall study this connection before commenting on the most efficient scheme to vary TGen and TVar.

### 3.4 Timing Analysis

**Figure 10:**
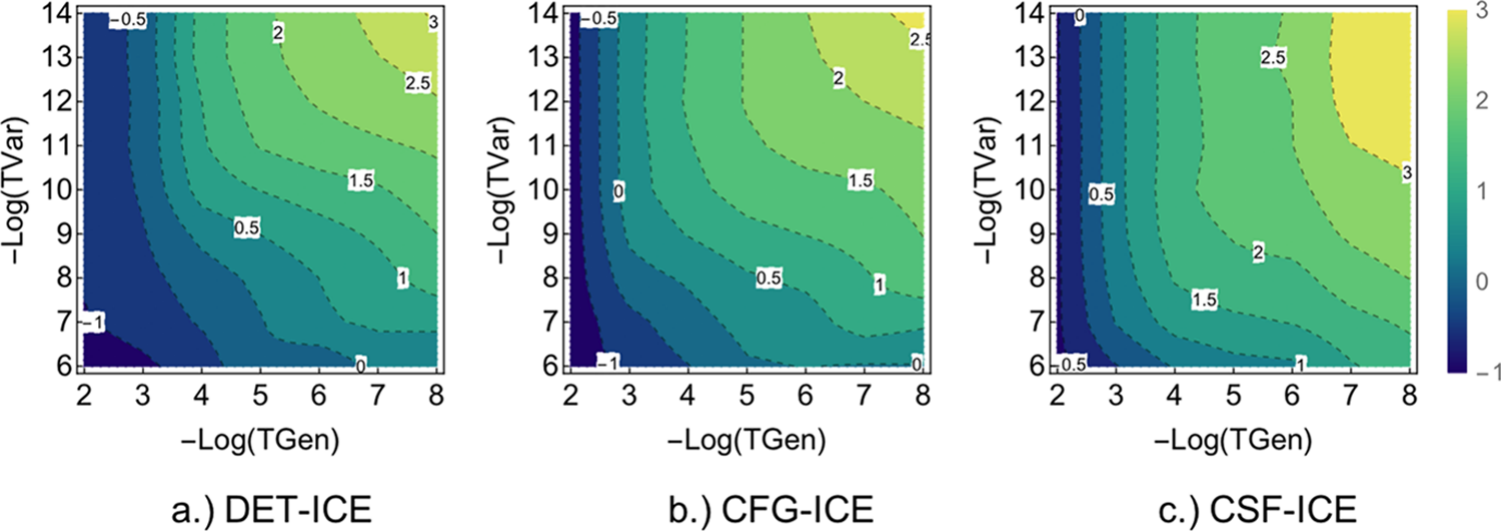
Timing comparison with respect to TGen and TVar for the NH_3_ molecule in the cc-pVDZ basis. Contour lines give the time in 10*^z^* minutes, where *z* is the label shown on the contour line.

The most time-consuming part of each ICE iteration is the Davidson diagonalization step as explained in Part I and shown in Figure 1. The diagonalization of the selected space is crucial to generate the next best wavefunction |Ψ^*k* + 1^⟩, which is expanded in the basis of the “selected” MPBFs. Therefore, the size of the selected space of MPBFs at each ICE step is directly related to the cost of the total calculation. As shown in the previous section, the total number of MPBFs increases with decreasing thresholds TGen and TVar; consequently, the cost of the calculation increases with decreasing TGen and TVar as illustrated for the NH_3_ molecule in double-ζ basis (as described in Section 3.1) in Figure 10. A more detailed analysis of the parallel scaling and computational efficiency on larger molecules will be given in Part III of the current series of papers.

Therefore, a cost-benefit analysis can help to understand the most effective path to systematically vary TGen and TVar value for which we get the best energy for the least computational time. This analysis will be performed in the following section for the three many-particle representations.

### 3.5 Optimal Thresholds

In order to be able to extrapolate energies obtained with progressively tighter thresholds, one needs to identify the optimal way to reduce the thresholds. This choice can be made by looking at the efficiency of an ICE calculation, which is dependent on the following two factors: First, the cost of the calculation, which is proportional to the number of wavefunction parameters needed to optimize. Second, the accuracy of the calculation obtained at a given TGen and TVar. A tighter choice of thresholds will permit a more accurate result while at the same time incurring a large cost of calculation. In order to obtain a “cost-benefit index” (CBindex), the function shown in eq 11 has been used:
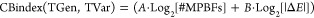
11where the two constants and serve to scale the cost due to the total number of MPBFs (#MPBFs) and the energy error (Δ*E*), respectively. Here the number of MPBFs in the calculation has been used as a proxy of the total computational time as described in Section 3.4. In the present case, *A* was set to 1 and *B* to 0.7 in order to obtain comparable magnitudes for CBindex.

**Figure 11:**
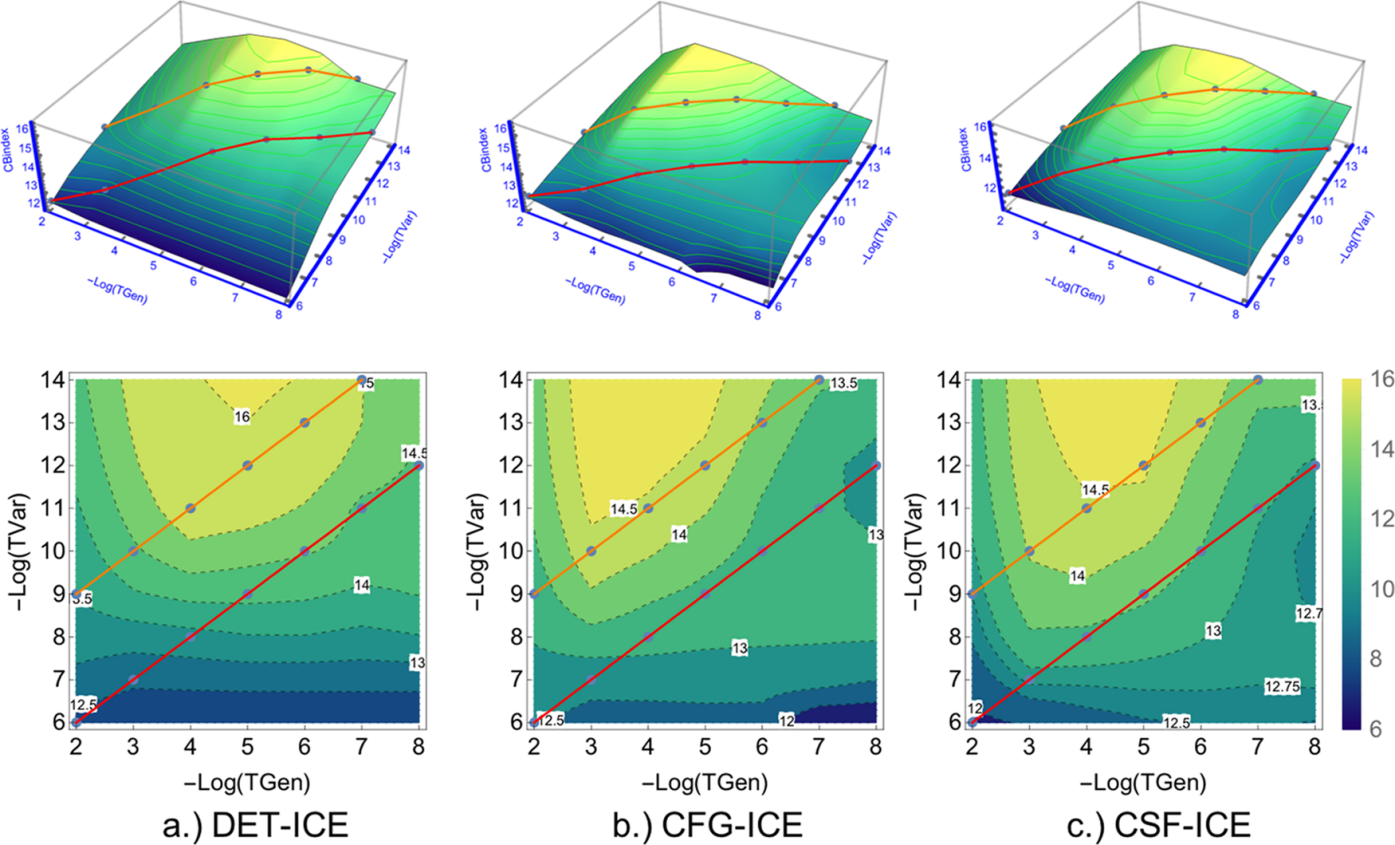
Cost-benefit index plot for the three types of the ICE vs the two thresholds TGen and TVar. Units are arbitrary but identical for the three plots. The paths show the most optimal way to increase the accuracy while keeping the cost of the calculation approximately constant. This corresponds to following paths in red and orange with τ = 3 and τ = 7, respectively, and decreasing TGen.

Using the function given in eq 11 we can plot the total CBindex versus the two thresholds TGen and TVar as Figure 11. There is a clear region of parameters (shown in darker colors), which has the best cost-benefit ratio. These regions are slightly different for the DET, CFG, and CSF-ICE. General trends can be seen from the above plots, which are the following:

a) In order to decrease the error, one would benefit from decreasing the TGen while keeping TVar fixed rather than the other way around. This is more important for smaller TGen thresholds. For example, in order to improve the error obtained at TGen = 10^–4^ and TVar = 10^–9^, it is more advantageous to decrease the TGen to 10^–6^ while keeping TVar constant (=10^–9^) than keeping TGen = 10^–4^ and decreasing TVar to 10^–11^. This manner of optimal change of parameters is shown visually with the two lines on the upper section of Figure 11 above.
b) In Figure 11, one can also see a region of constant cost-benefit value with decreasing TGen and a fixed TVar. This implies that the increase in the wavefunction parameters (due to decreasing TGen) is compensated for by a more accurate energy. This suggests an efficient protocol for obtaining better energies at minimal cost, which shall be described below.
c) For small molecules, it is convenient to use a single parameter τ (see Section 2.1) such that the energies are converged with respect to TVar, such as τ = 7 and a small enough value for TGen=10^–4^ to achieve CCSDT(Q) quality results as will be described below in more detail.
d) In practice, for larger molecules, the optimal thresholds seems to be to fix τ to a small value such as τ = 3 (i.e., TGen/TVar =10^3^) or τ = 4 (i.e., TGen/TVar =10^4^) and decrease TGen alone. This strategy leads to the smallest computational cost while at the same time obtaining the best possible total energy. This will be discussed in more detail in Section 3.7.

Given that these variations are highly systematic, an extrapolation scheme to recover the FCI energy from successive calculations with tighter thresholds should be computationally attractive. We will investigate this subject in the next section after a brief summary of the threshold defaults extracted from the benchmarking analysis.

### 3.6 Summary of Benchmarking Analysis

The ORCA default since 2015 has been ICE(4,7), which leads to results that are better than CCSDT(Q) quality. In these calculations due to the small value of τ, the rest energy is so small that adding it to the variational energy leads to insignificant changes. Notice that the setting with ICE(4,3) provides similar results to the much tighter ICE(3,7) setting at a much lower computational cost (c.f. Table 3, timings). Therefore, for molecules with larger FCI spaces, thresholds with τ = 3 and successively smaller TGen values combined with an extrapolation scheme might be a better choice. This will be analyzed in more detail in Section 3.7. Finally, we would like to point out that with ICE(2, 7) or ICE(3, 3), we obtain results that are comparable to CCSD(T) quality, while with ICE(3, 7) or ICE(4, 3) CCSDT(Q) quality is reached. Further tightening the thresholds to ICE(4, 7) surpasses the accuracy of CCSDT(Q) results for the FCI21 benchmark set.

**Table 3:**
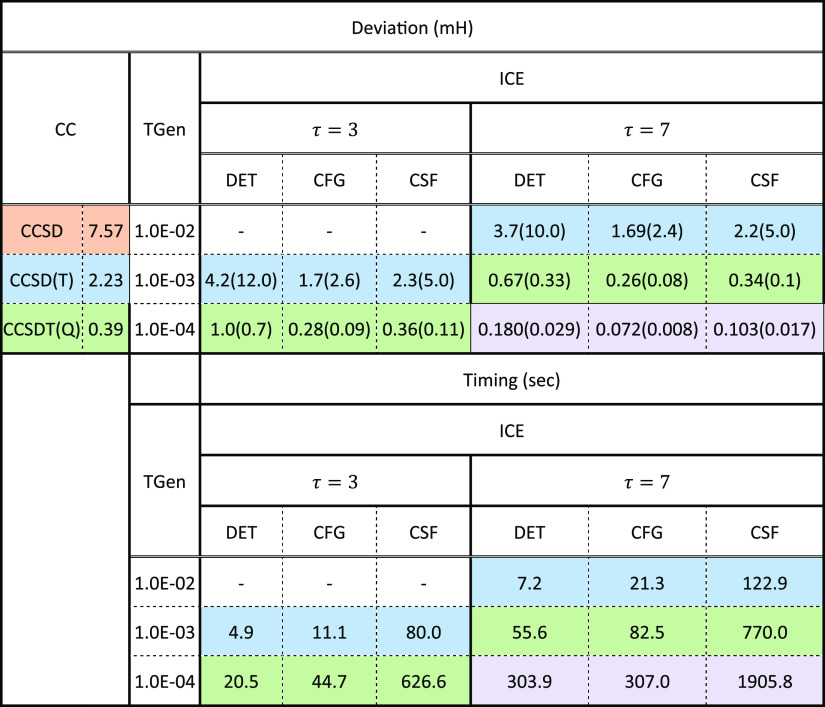
Comparison of the Energy Deviation for ICE with Various Threshold Values (TGen) and Ratios (τ) and Coupled Cluster (CC) with Respect to FCI Energies^a^

a The corresponding timings for obtaining the errors are also given in seconds. The colors show comparable error range for the ICE and CC calculations. Numbers in parentheses beside the errors give the variance with respect to the FCI21 set.

### 3.7 Extrapolation Scheme

#### 3.7.1 Extrapolation Protocol

As the objective of a sCI calculation is to approximate the FCI energy as closely as possible and any sCI method must introduce truncation thresholds, it is tempting to devise extrapolation schemes that allow one to estimate the FCI energy obtained at zero threshold. Such an extrapolation scheme has been studied in great detail by Buenker and Peyerimhoff in the early 1970s^41,42^ and by Angeli and Persico et al. in a series of papers in the late 90s.^39,43,44^ Since these pioneering contributions, a number of extrapolation schemes have appeared in the literature for sCI methods, which can be divided into two types: (1) the extrapolation with weight of the generator coefficients (or equivalently generator thresholds) as done by the original papers by Buenker and Peyerimhoff and by Angeli and Persico et al. and others^18,43−46^ and (2) extrapolation with respect to the PT2 energy correction due to the rejected configurations as done, e.g., by Holmes et al.^47,48^ Inspired by the later studies, we have followed a slightly modified approach here.

**Figure 12:**
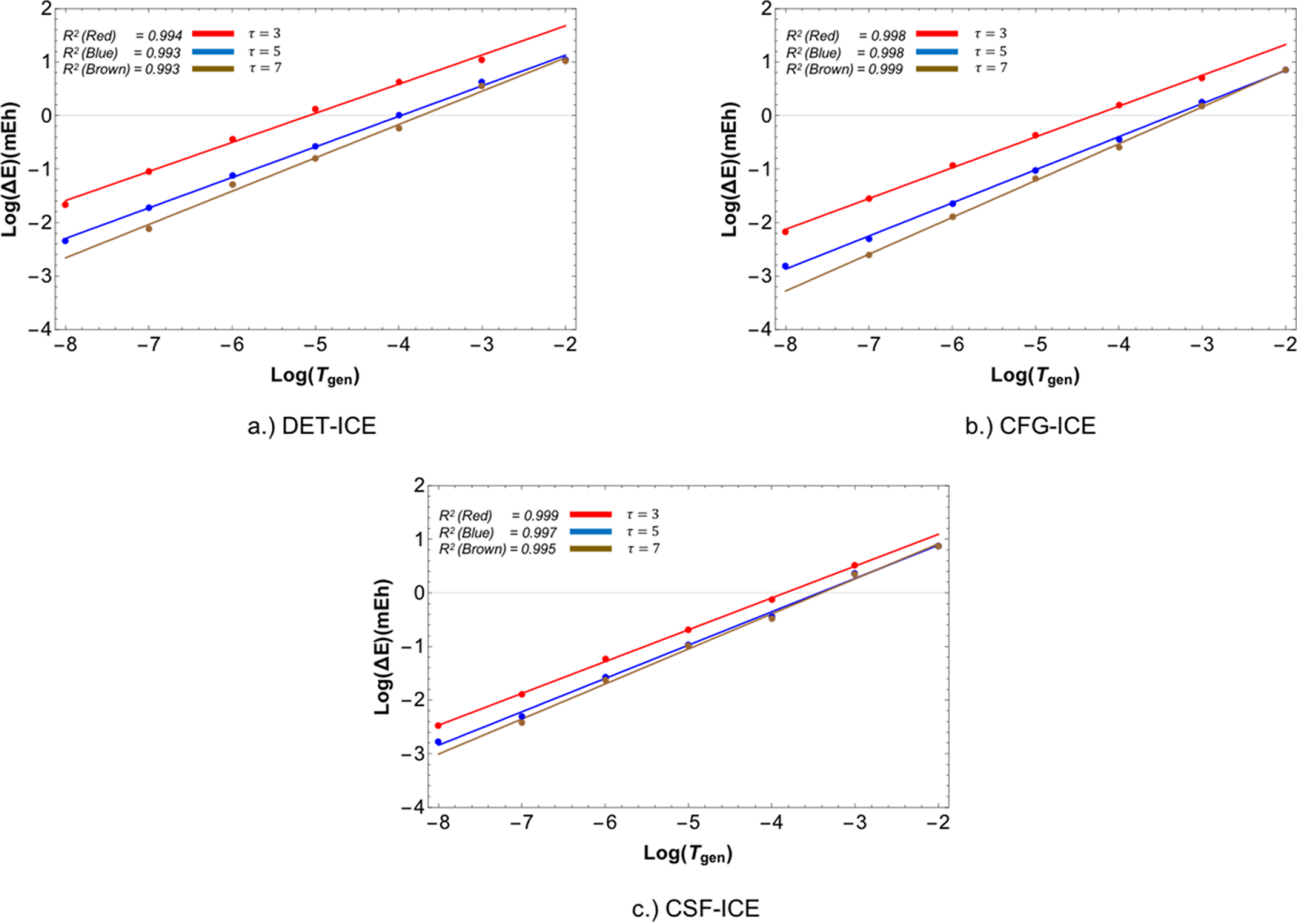
Extrapolation of the error (vs FCI) with respect to the generator threshold TGen. The three ratios τ = 3,5, and 7 are shown in red, blue, and brown, respectively, to illustrate the exponential convergence in the two cases. The *R*^2^ value gives the quality of a straight line fit to the data, which corresponds to the FCI21 benchmark set.

In our case, there are two thresholds TGen and TVar. However, as pointed out above below, one can devise a composite threshold by fixing the ratio of TGen/TVar represented by τ and varying only TGen. Once a composite parameter (τ) is chosen, the energies can be extrapolated against TGen (this scheme is represented as ICE(−Log_10_(TGen), τ) as explained in Section 2.1). Here, we chose TGen with a fixed τ as the unique parameter for performing the extrapolation. As born out by our calculations (*vide infra*), the convergence of the correlation energy tends to be exponential with respect to the threshold TGen (keeping τ fixed) shown Figure 12. In fact, our observation that the energies can be extrapolated as a function of the weight of the generators is in agreement with the work by Angeli et al.^40^ and others.

Moreover, by design of the ICE algorithm, the error in the energy decreases with a decrease in the PT2 contribution of the rejected MPBFs (*E*_*PT*2_) known as the “rest” energy. This is in agreement with the finding that the energies can be extrapolated against the “rest” PT2 energy (*E*_*PT*2_) with the extrapolated value (*E*_*PT*2_^∞^) obtained as *E*_*PT*2_ → 0. Moreover, it has been realized that an extrapolation of the energy with the “rest” PT2 contribution tends to be more linear than the extrapolation relative to the thresholds.^47^ Therefore, we use a simple (two-point) linear function to estimate the extrapolated energy as a function of *E*_*PT*2_ as shown in eq 12 below:
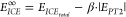
12

The parameters *E*_ICE_^∞^ and β are obtained by a fit to the data obtained by varying TGen for a fixed τ. Here, *E_ICE_total__* represents the total energy, i.e., *E_ICE_total__* = *E_ICE_var__* + *E*_*PT*2_. Obviously, two calculations are enough to determine the parameter β. However, successive data points can be added to continuously improve the fit. With three calculations (three-point fit) or more, one can also obtain the confidence interval (Δ*E*_ICE_^∞^) of the extrapolated energy as shown in eq 13:
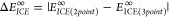
13

Upon convergence or close to convergence, a two-point extrapolated energy (*E*_ICE(2*point*)_^∞^) and three-point extrapolated energy (*E*_ICE(3*point*)_^∞^) will be very close and hence Δ*E*_ICE_^∞^ will be close to 0. Thus, a confidence interval of Δ*E*_ICE_^∞^ = 0 will imply a high confidence of the extrapolation for small enough TGen and τ values. Note that this extrapolation scheme is general and transferable for any MPBF and essentially comes without additional overhead or changes to the ICE algorithm.

#### 3.7.2 Application on the FCI21 Data Set

**Figure 13:**
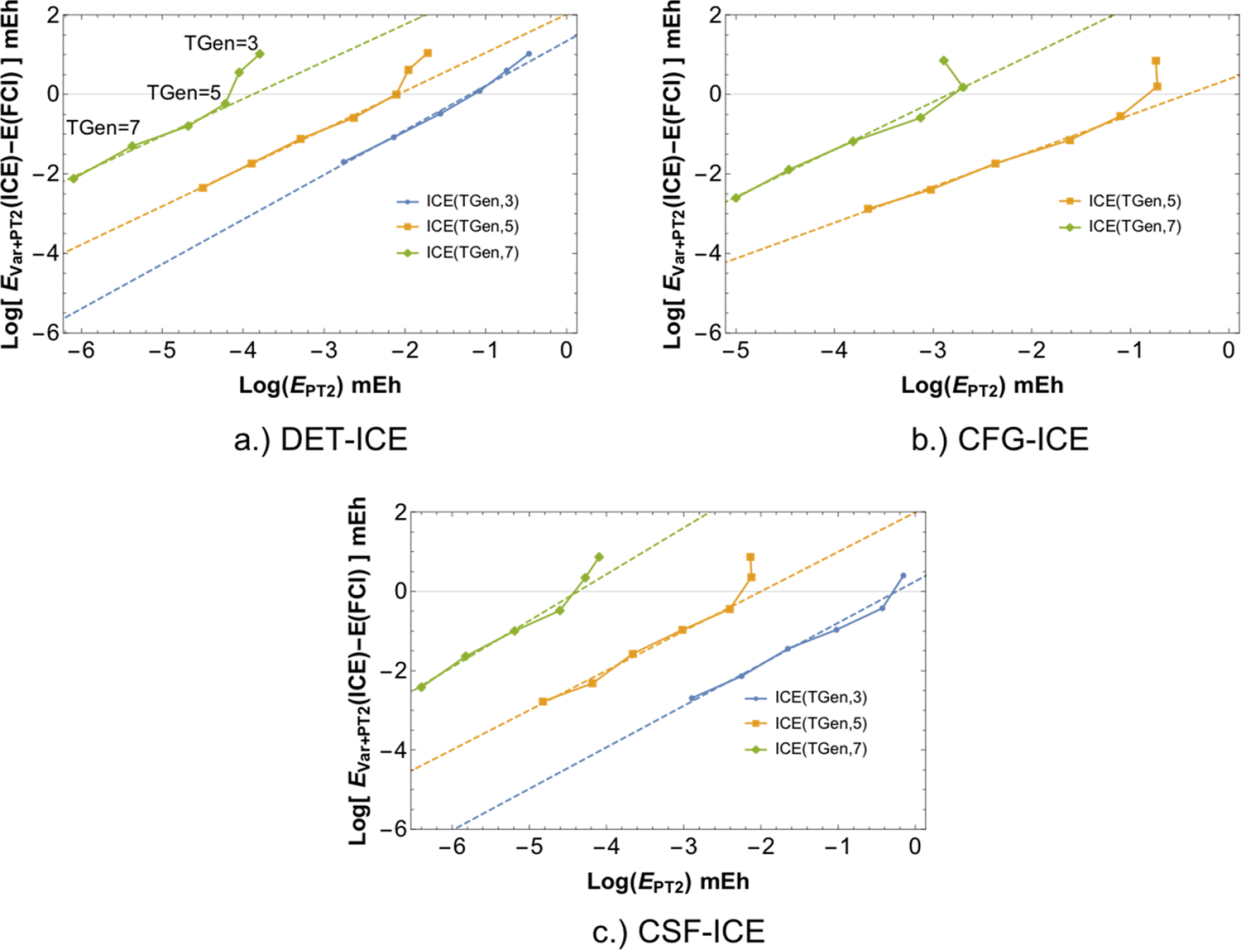
Convergence of the error in energy (relative to FCI) vs the PT2 “rest” energy (*E*_*PT*2_). The four ICE protocols used are compared with each other for all three MPBFs. A linear fit to the data is shown in dashed lines.

As an illustration of our extrapolation scheme, we plot the energy convergence of the FCI21 set with a series of calculations ICE(*A*, τ) (with τ = 3,4,5, and 6) . As shown in Figure 13, the total energy converges linearly as a function of the *E*_*PT*2_ toward the FCI energy for all four schemes ICE(*A*,3), ICE(*A*,4), ICE(*A*,5), and ICE(*A*,6).

A linear fit to obtain the extrapolated energy *E*_ICE_^∞^ can be obtained by a choice of two calculations with TGen =*m* and TGen =*n* for a given fixed τ, which we shall denote by *EP*(*m*/*n*, *τ*) – *ICE*. A three-point extrapolation would be denoted as *EP*(*m*/*n*/*k*, τ) – *ICE*.

In order to access the accuracy of the extrapolated energy *E*_ICE_^∞^ with a two-point fit, we have performed an analysis of a series of systematic choices *EP*(*m*/*n*, τ) – *ICE* for the FCI21 benchmark set.

The series of extrapolated energies with *m* = 4 to *m* = 7 show a monotonically increase in accuracy for the FCI21 set as shown in Table 4. It is clear from Table 4 that the average absolute error (including the variance) of the extrapolated ICE energies with τ = 3 and τ = 7 are smaller than 1 mH for all the molecules in the FCI21 irrespective of the two points chosen *EP*(*m*/*n*). However, the accuracy of the extrapolated energy increases with decreasing TGen, as expected. Note that the calculations with τ = 3 are at least an order of magnitude cheaper than that with τ = 7. However, the quality of the extrapolated energies with τ = 7 is better than that with τ = 3.

**Table 4:** Comparison of the Two-Point Extrapolation Scheme EP(*m*/*n*, τ) with τ = 3 and τ = 7 and the ICE(TGen,τ) Single-Point Energies^a^

	error (mH)				
	extrapolated			E(Var+PT2)	
	τ			τ	
EP(*m*/*n*)	3	7	ICE(TGen,*τ*)	3	7
(4/5)	0.080(0.130)	0.040(0.050)	5	0.800(1.100)	0.070(0.080)
(5/6)	0.018(0.025)	0.005(0.012)	6	0.180(0.260)	0.013(0.016)
(6/7)	0.004(0.005)	0.004(0.006)	7	0.030(0.040)	0.002(0.005)
(7/8)	0.002(0.004)		8	0.007(0.009)	

	cost (seconds)				
	extrapolated			E(Var+PT2)	
	τ			τ	
EP(*m*/*n*)	3	7	ICE(TGen,*τ*)	3	7
(4/5)	898.5	6312.7	5	704.7	4407.0
(5/6)	2639.6	22112.7	6	1934.8	17705.7
(6/7)	7911.0	66786.0	7	5976.2	49080.3
(7/8)	27694.0		8	21717.8	

a The FCI21 data has been used to compute the error (in mH) between the FCI energy and the ICE/two-point EP(*m*/*n*,τ) extrapolated energies. The variance of the computed error (i.e., error bars) are given in parentheses. Timings correspond to the same FCI21 set. All results are for the CSF-ICE variant.

As seen from Table 4, the two-point extrapolation scheme (at least with τ = 3) results in errors, which are about an order or magnitude smaller than the single-point ICE(TGen,τ) calculation. Moreover, this improved error comes at a similar cost as the parent ICE(TGen,τ) calculation with the larger TGen. Therefore, a two-point extrapolation scheme with τ = 3 is a viable option, which provides much improved energy estimates at essentially negligible additional cost.

It is now tempting to test this for larger systems for which FCI values cannot be obtained and compare the extrapolated FCI energies to coupled-cluster ones (CCSDT(Q)) or DMRG energies as will be done in the next section.

#### 3.7.3 Application of Extrapolation Scheme

**Figure 14:**
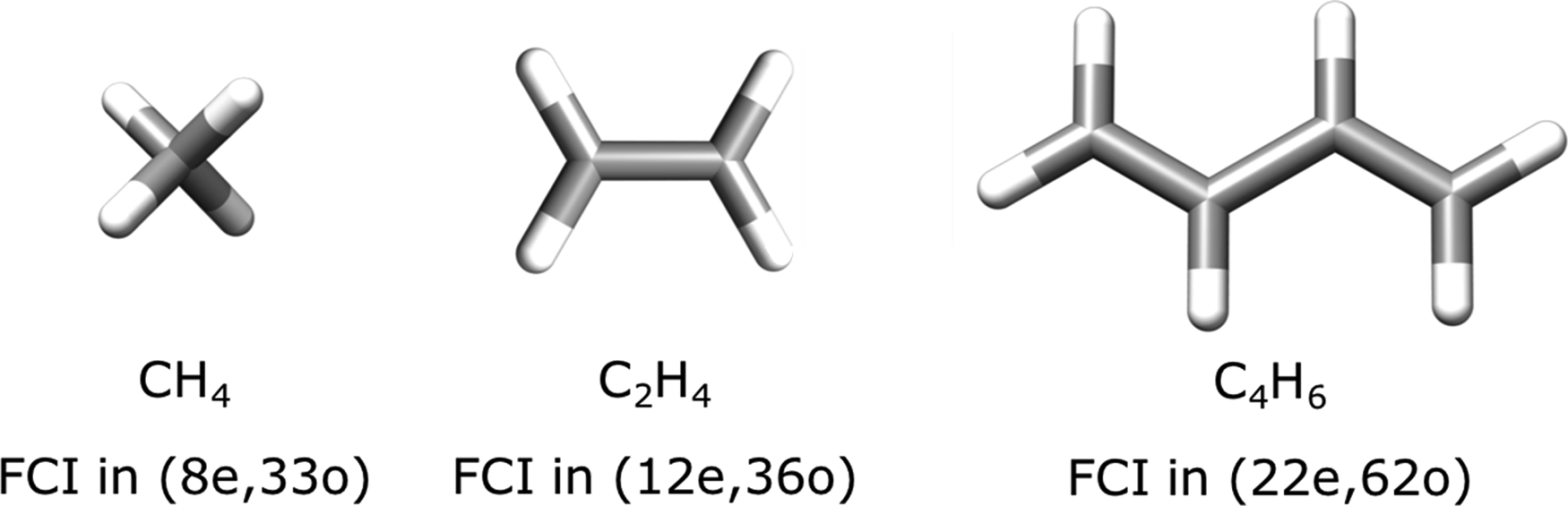
Three model systems used to check for the proposed extrapolation scheme proposed in Section 3.5.

In order to test the extrapolation scheme proposed in the previous section, we carry out ICE calculations on three small polyenes in a double ζ-basis set as shown in Figure 14 below.

The cc-pVDZ basis set is used for hydrogen atoms, and the SV basis set is chosen for the carbon atoms for C_2_H_4_ and C_4_H_6_. The cc-pVDZ basis was used for all atoms for the case of methane (CH_4_). The geometries of the three molecules are given in Section 1.4.1 of the Supporting Information.

##### 3.7.3.1 Detailed Study of Ethene Molecule

In order to illustrate the quality of the linear fit given in eq 12 for different values of τ and to demonstrate the extrapolation procedure, we first do an exhaustive study on the ethene molecule. The ICE calculation and extrapolation of to obtain the FCI energies has been done as follows:

a) The scheme ICE(TGen, τ) has been used for all the three molecules with TGen =3,4,5,6,7, and 8 where possible.
b) Once the series of energies has been obtained, an extrapolated FCI energy is chosen (*E*_ICE_^∞^) by fitting to *E*_*PT*2_ with a straight line as we have shown for the benchmark set. This gives a unique value of the extrapolated energy *E*_ICE_^∞^, which can be obtained by setting *E*_*PT*2_ → 0 in eq 12.

Note that, close to convergence, a confidence interval can be extracted by using three best lowest energies and comparing the extrapolated energies obtained by the two-point fit and the three-point fit as described in Section 3.7.1.

**Figure 15:**
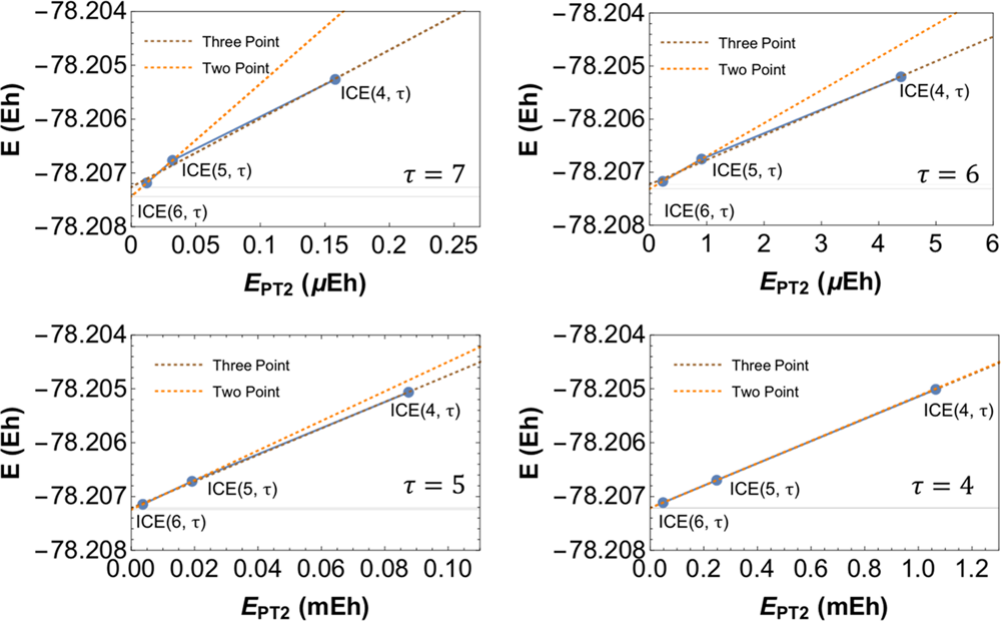
Demonstration of the linear fit procedure with respect to the “rest” PT2 energy. Four values of the combined threshold τ have been used to illustrate the linearity as a function of τ and TGen parameters. Data is taken from CSF-ICE calculations on the ethene molecule with double-ζ basis with a FCI space of (12e,36o).

The extrapolation for four values of τ (4,5,6,7) is shown in Figure 15. The corresponding extrapolated energy as compared to CCSDT(Q), and confidence intervals is given in Table 5. As seen from Figure 15 and Table 5, the extrapolation can be made with a linear fit of the “rest” PT2 energy for a given τ. Smaller values than τ = 3 give a better linear fit with decreasing TGen, which is encouraging as they also correspond to a more compact wavefunction (c.f. Table 5). Therefore, a two-point fit can give reliable extrapolated energies (<1 mH).

**Table 5:** Comparison of the Extrapolated Energy Error (vs CCSDT(Q)) and Confidence Interval for Various Values of τ for CSF-ICE^a^

τ	*E*_*ICE*(6, *τ*)_	*E*_ICE_^∞^	error (mEh)	% NCSFs
7	–78.207186	–78.2073(2)	0.6(2)	100.0
6	–78.207172	–78.20723(8)	0.64(8)	89.1
5	–78.207142	–78.20722(3)	0.66(3)	71.1
4	–78.207066	–78.207213(1)	0.658(1)	49.0
3	–78.206841	–78.2074498	0.450	31.8

a A comparison of the CSFs (vs the largest calculation τ = 7, NCSF =3793459) is also given in order to compare the compactness of the wavefunction. The data is for the C_2_H_4_ molecule with double-ζ basis and a FCI space of (12e, 36o). The best variational energy *E*_*ICE*(*TGen*, *τ*)_ for each *τ* is also given for comparison. The τ = 3 extrapolated energy is taken from the three-point formula.

##### 3.7.3.2 Comparison with All Molecules

**Figure 16:**
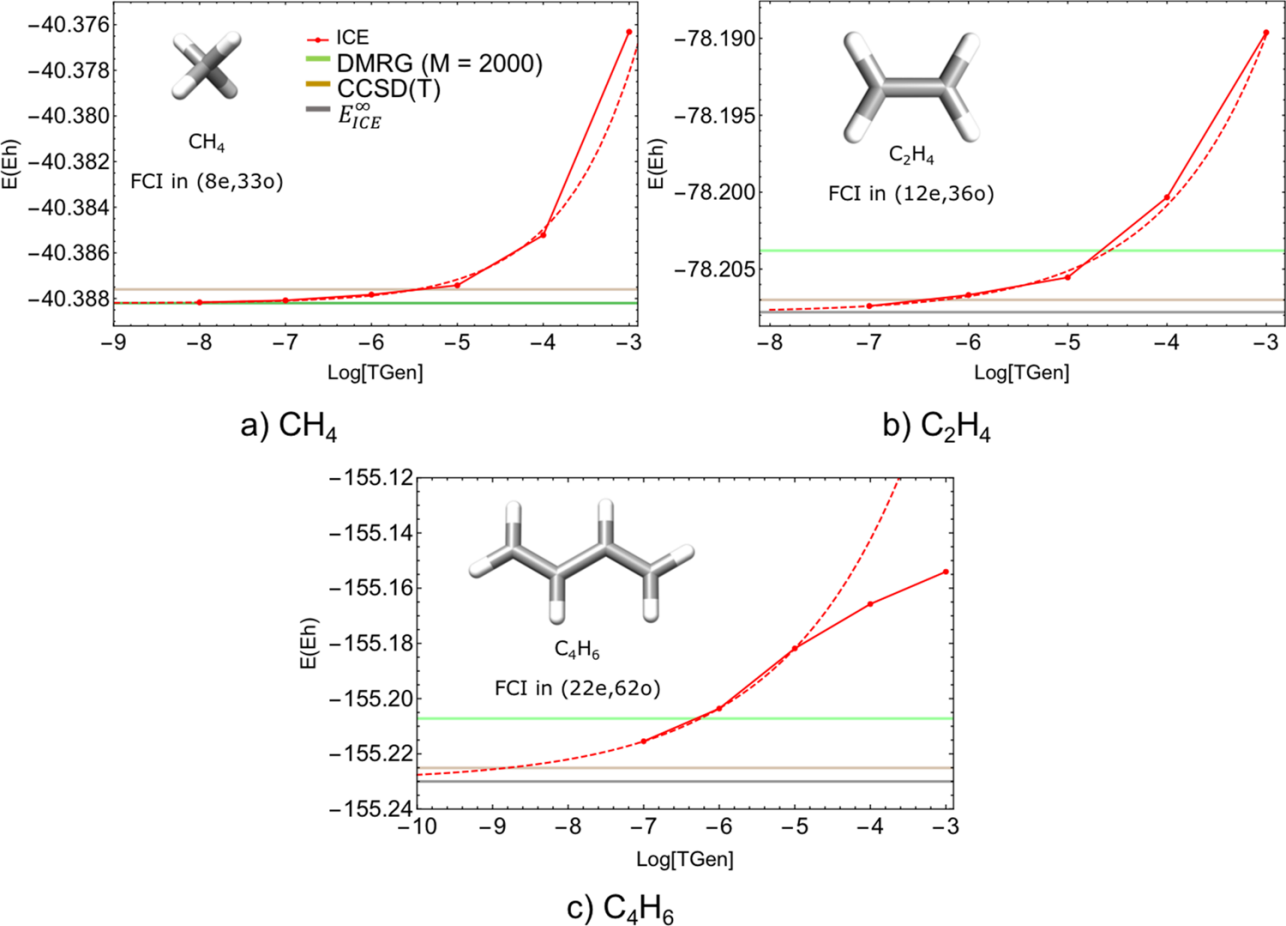
Extrapolated energy for the three conjugated polyene molecules (CH_4_, C_2_H_4_, and C_4_H_6_) along with the DMRG and coupled-cluster energies. The cc-pVDZ basis is used for hydrogen atoms and SV basis for carbon atoms. The correlated number of electrons and orbitals for each system is shown as an inset. The ICE(TGen,τ = 3) protocol (i.e., TVar = TGen·10^–3^) is used for all points.

Now we can proceed to the results for the three molecules together. These three molecules are too large for an exact FCI calculation in the given basis set and hence comparison cannot be directly made with FCI results. Nevertheless, one can obtain near-FCI quality energy for such closed shell molecules from CCSDT(Q) calculations. First, we compare the convergence of the energies as a function of the threshold TGen keeping τ = 3, i.e., a scheme with ICE(TGen, 3) for decreasing values of TGen as shown in Figure 16. The smallest feasible TGen value was 10^–8^ for CH_4_ and 10^–7^ for C_2_H_4_ and C_4_H_6_, which we used to obtain the best variational energies, respectively. As can be seen from Figure 16, convergence is exponential for all three molecules and the energies of methane and ethene are well converged and lower than the CCSD(T) energies.

Following the convergence of the pure variational energy, one can proceed to the extrapolation. The variational and extrapolated ICE, CCSDT(Q), and DMRG energies are shown in Table 6 below.

**Table 6:** Comparison of the Correlation Energies (in Millihartree) Obtained by CCSDT(Q), ICE, and DMRG for the Three Small Polyenes^a^

			DMRG (MaxM)		ICE		
	size	CCSDT(Q)	1000	2000	EP(*m*/*n*,3)	variational	extrapolated
CH_4_	(8e,33o)	189.9	189.9	190.0	(7, 8)	189.8	189.8
C_2_H_4_	(12e,36o)	250.3	245.6	246.1	(6, 7)	249.8	249.8
C_4_H_6_	(22e,62o)	464.9	438.1	438.7	(6, 7)	453.6	460.2

a A two-point scheme has EP(*m*/*n*,3) has been used for the extrapolation. The variational energies correspond to the most accurate calculation, i.e., ICE(*n*,3) for each molecule.

The quality of the extrapolated energies given in Table 6 can be compared with the energy convergence with TGen shown in Figure 16. The convergence of the extrapolated energies is shown in Figure 11 of the Supporting Information. The difference between the *E*_ICE_^∞^ obtained by progressively adding more accurate *E_ICE_*, and *E*_*PT*2_ values can give reliable confidence intervals for the extrapolated FCI energy *E*_ICE_^∞^.

The main point to emphasize is that the variational and extrapolated energies are very close to the CCSD(T) energies for all molecules and CCSDT(Q) for CH_4_. In all cases, the variational energies are lower than the DMRG estimates and even the CCSD(T) energies for CH_4_ and C_2_H_4_ as shown in Figure 16. The extrapolated energies are always lower than the DMRG energies and are very close to the CCSDT(Q) values. Therefore, this manner of extrapolation is an effective and transferable choice for moderate to large size molecules and can give energies comparable to the CCSD(T) values at least for the systems studied here.

#### 3.7.4 Summary of the Extrapolation Analysis

In conclusion, a judicious choice of the extrapolation scheme EP(*m*/*n*, *τ*) can be used to systematically reduce the error by at least an order of magnitude with respect to the variational ICE value. We have observed that, for small molecules, i.e., about 14 electrons, a tighter scheme with τ = 4 or τ = 5 can give an accurate approximation (less than 0.1 mEh error) to FCI as shown in Figure 13. However, for larger molecules with more than 30 electrons, such tight thresholds are not feasible. In such cases, an EP(*m*/*n*, τ) scheme with τ = 3 can be a viable alternative to get at least results of about CCSD(T) quality. However, unlike the latter, the ICE is not restricted to single reference systems. Moreover, we clearly acknowledge that the energies obtained here suffer from size-inconsistency errors, which is the primary reason behind the lack of accuracy with respect to CCSDT(Q) values. This size-inconsistency error will be studied in the next section.

### 3.8 Size-Inconsistency Error

The size-inconsistency error (SIE) associated with the ICE method arises due to the truncated CI expansion and contributes to the deviation from the FCI energy. For a pedagogical and clear description of the SIE for approximate CI methods, see the description by Malrieu et al.^49^ Recently, an analysis of the SIE for sCI methods using DET MPBF has been carried out by Ten-no et al.^50^ Here, we shall use two examples to illustrate and compare the SIE for the three MPBFs used in the present work.

#### 3.8.1 Neon Dimer

The SIE in the present formulation of the DET, CFG, and CSF-ICE can be demonstrated using the neon dimer. In order to calculate the size-inconsistency error, we proceed as follows: the FCI energy of the Ne atom in the cc-pVDZ basis with (8e,8o) (with a frozen core) is calculated followed by the energy of the Ne dimer (16e,16o) at a 10 Å distance. The SIE can then be calculated by subtracting twice the neon energy from that of the neon dimer as shown in q 14:
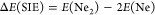
14

**Figure 17:**
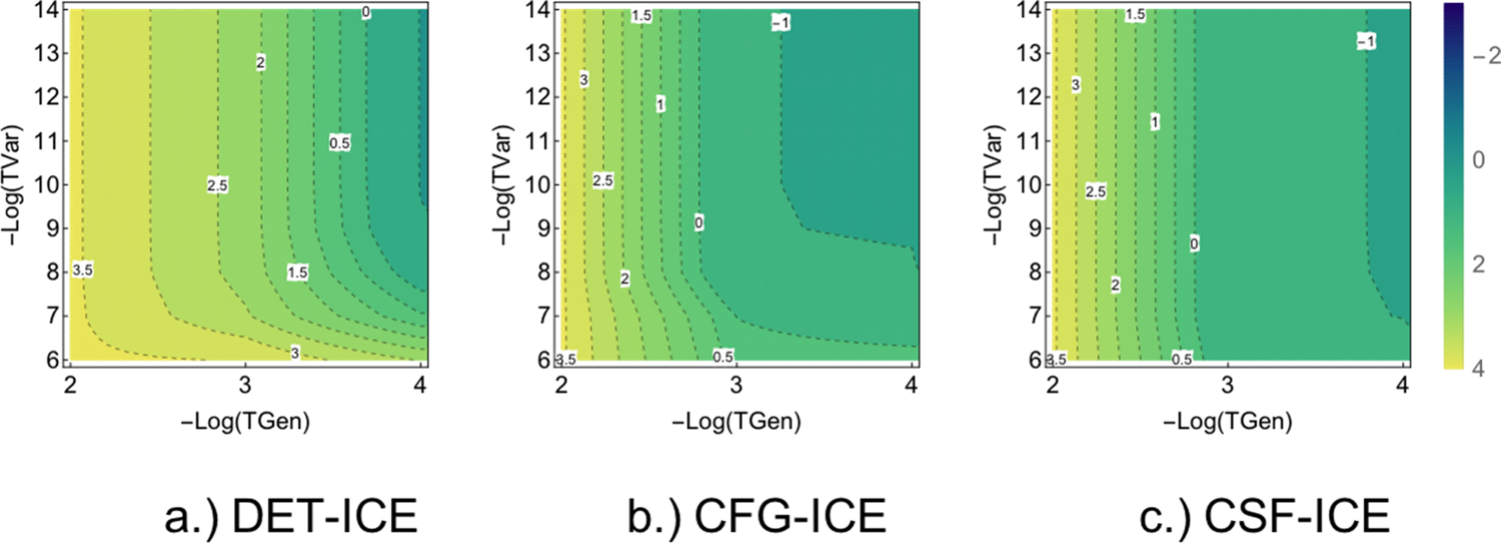
SIE for the neon dimer separated by 10 Å, as a function of the two thresholds TGen and TVar. The SIE is calculated by taking the difference between the energy of the Ne_2_ molecule and twice the energy of the Ne atom.

For a size-consistent method, this difference should vanish since the distance between the Neon atoms (10 Å) makes it an essentially a non-interacting pair of atoms. A deviation from zero therefore results from the size-inconsistency error introduced by the truncation of the CI space. The error in as a function of TGen and TVar is shown in Figure 17. Note that when TGen and TVar go to zero, the wavefunction approaches the FCI one and therefore the size-inconsistency error vanishes, as expected. From this illustrative example, it becomes clear that the SIE is of the same order of magnitude as the total error (see Figure 3) and has to be analyzed for larger molecules. This shall be done in the next section.

#### 3.8.2 Ethene + Neon Atom

**Figure 18:**
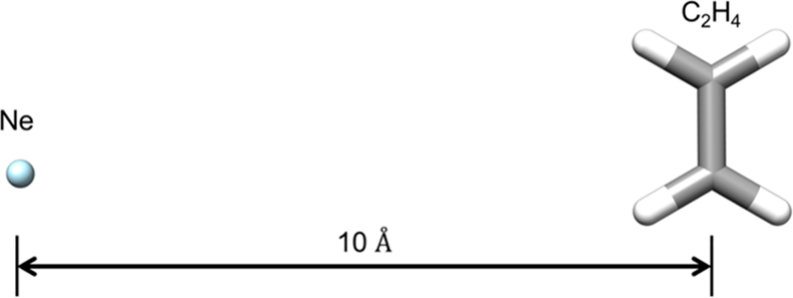
Ethene and neon example used to examine the size-inconsistency error as a function of ICE thresholds.

A more rigorous test of the SIE can be performed using a larger system consisting of the ethene molecule with a neon atom separated by 10 Å, thus ensuring that the two molecules are essentially non-interacting (Figure 18). The cc-pVDZ basis is used for Ne (8e,8o) and H, whereas the SV basis is used for the carbon atoms; thus, the FCI space consists of (20e, 44o) for the combined system.

A size-consistent method will give total energies, which can be exactly written as a sum of the energies of the ethene and Ne molecules separately. Therefore, the SIE can be obtained by subtracting the FCI energy (or extrapolated FCI energy in the case of ethene) of ethene and Ne molecules calculated separately from the ICE calculating involving the full system as given in eq 15:
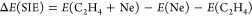
15

**Figure 19:**
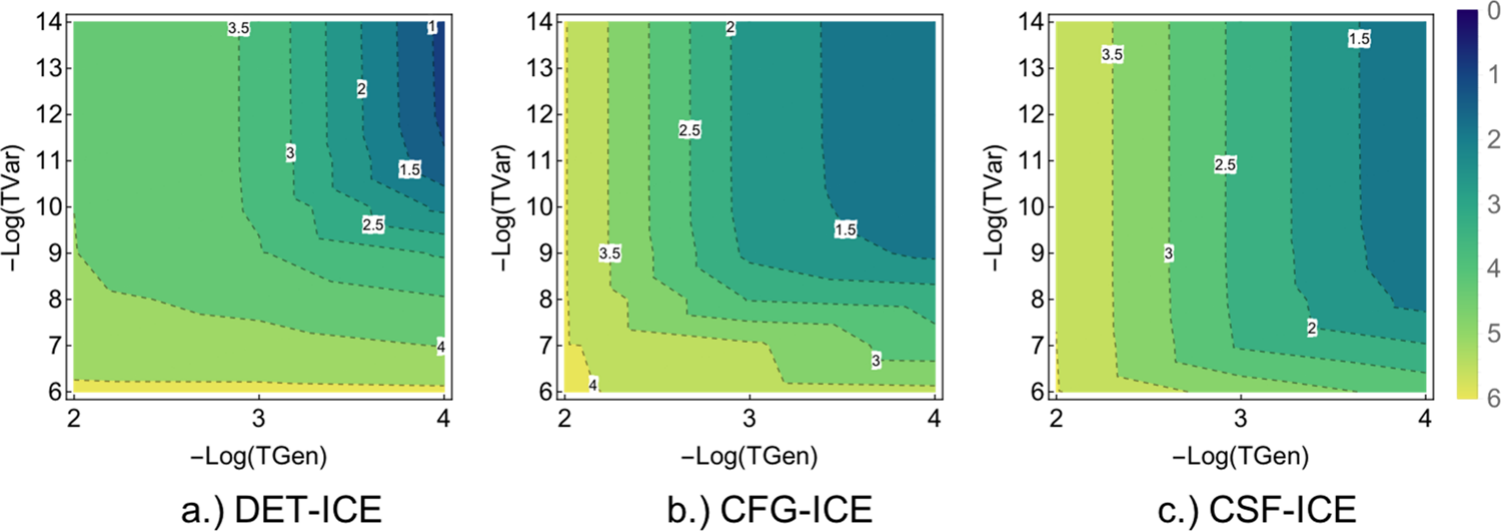
Comparison of the SIE for the ethene and neon system with the neon atom at about 10 Å distance. The error is in millihartree and is given as Log_2_(Δ*E*(*ICE*) – *E*(*ICE*^∞^)], where *E*(*ICE*^∞^) is the extrapolated approximate FCI energy for ethene and the exact FCI energy for the neon system summed together.

The similarity of Figures 17 and 19 with those showing the total energy error (Figure 3) indicate that the size-inconsistency word error is the main ingredient missing from such selected CI calculations. Notice also that the magnitude of the SIE for Ne_2_ is almost equal to the magnitude of the total deviation from the FCI energy of the benchmark set as shown in Figure 3, suggesting that a large part of this deviation originates from the SIE. Therefore, it makes it imperative to formulate a correction of the size-inconsistency error in order to improve the accuracy of the present selected CI scheme. This is currently a work in progress in our laboratory.

## 4 Conclusions

In this second part of our three series of papers, which aims a comparison of the three many-particle representations, we have performed a benchmark test of comparison of the three methods with FCI for a set of 21 diatomic molecules. There are some interesting conclusions that emerge from the present analysis:

a) Due to the spin-adapted formalism of the CFG-ICE and CSF-ICE, these two many-particle bases lead to a significantly more compact many particle wavefunction than the DET-ICE many-particle basis representation. This allows for a better accuracy with a similar number of wavefunction parameters. This will be capitalized on in much greater detail in concrete chemical applications in Part III of the series.
b) The behavior of the perturbative energy contribution for the CFG and CSF-ICE is quite different from the DET-based ICE. This has been demonstrated by the comparison of the spread of the PT2 energy contribution for the three MPBFs for the butadiene molecule. The main conclusion from this analysis is that the PT2 energy is spread out over about 1 order of magnitude more DETs compared to CSFs. Due to this larger spread of the PT2 energy contribution, the amount of PT2 energy brought in by one CSFs is about four times larger than that brought in by one DET. Therefore, even for the perturbative energy calculation, the CSF many-particle basis is more compact than the DET basis.
c) From our benchmark FCI21 set, the default setting of τ = 7 and TGen =10^–4^ reproduces 99.8% of the correlation energy, which is better than the CCSD(T) result with only 2% of the total time of a FCI calculation.
d) Based on the cost-benefit analysis, an extrapolation scheme was devised, which was shown to be effective for the benchmark set. We recommend different settings for high-accuracy studies on small molecules and studies on larger systems. For small systems, we find that ICE(4,7) provides results that surpass CCSDT(Q) quality, which is considered to be a converged level of theory. For larger molecules, compromises have to be made and such large τ values are not feasible. Here, we recommend τ = 3 together with a decreased TGen value of 10^–4^, which leads to excellent results and an optimal cost/benefit ratio. Relaxing these thresholds further comes at the expense of additional penalties in accuracy. However, depending on the application, slightly reduced accuracy may still be acceptable.
e) An open-ended linear extrapolation scheme was used to reduce the residual error of the calculation while not increasing the computational cost. Based on our results, a two-point extrapolation EP(3/4,τ) leads to a reduction in the error relative to ICE(4,τ) of one order of magnitude (FCI21 set) while still being no more expensive than the ICE(4,τ) calculation itself. Again, for larger number of correlated electrons, some compromises have to be made.
f) For larger systems, we have observed significant errors in the ICE final energies that we mainly attribute to the size-inconsistency inherent in the CI procedure. We have quantified these errors using the neon dimer and the ethene/neon molecule.

In the third part of the present series of papers, we shall perform case studies on different types of molecules in order to shed light on the strengths and weaknesses of the three types of many-particle basis representations.

## Supporting Information Available

Figures showing a detailed comparison of benchmark ICE results on the FCI21 set, geometry, and supplementary figures for the extrapolation analysis and detailed figures on the size-inconsistency error (PDF)
