# Supplementary material for: Comparison of Many-Particle Representations for Selected Configuration Interaction: II. Numerical Benchmark Calculations
Source: J Chem Theory Comput. 2021 Apr 22;17(5):2868–85. doi: 10.1021/acs.jctc.1c00081 (PMC8279407; doi:10.1021/acs.jctc.1c00081)
Supplement: Supplementary file 1 — ct1c00081_si_001.pdf [file ct1c00081_si_001.pdf]

Supplementary Information: Comparison of Many-Particle Representations for selected-CI II: Numerical benchmark calculations: Supplementary Information

Vijay Gopal Chilkuri, Frank Neese

Max-Planck-Institut für Kohlenforschung, Kaiser-Wilhelm-Platz 1, Mülheim an der Ruhr - 45470, Germany.

1.1 Comparison of variational ICE vs FCI energy

In this section we give detailed Figures with the error and variance for the FCI21 set with changes in TGen and TVar.

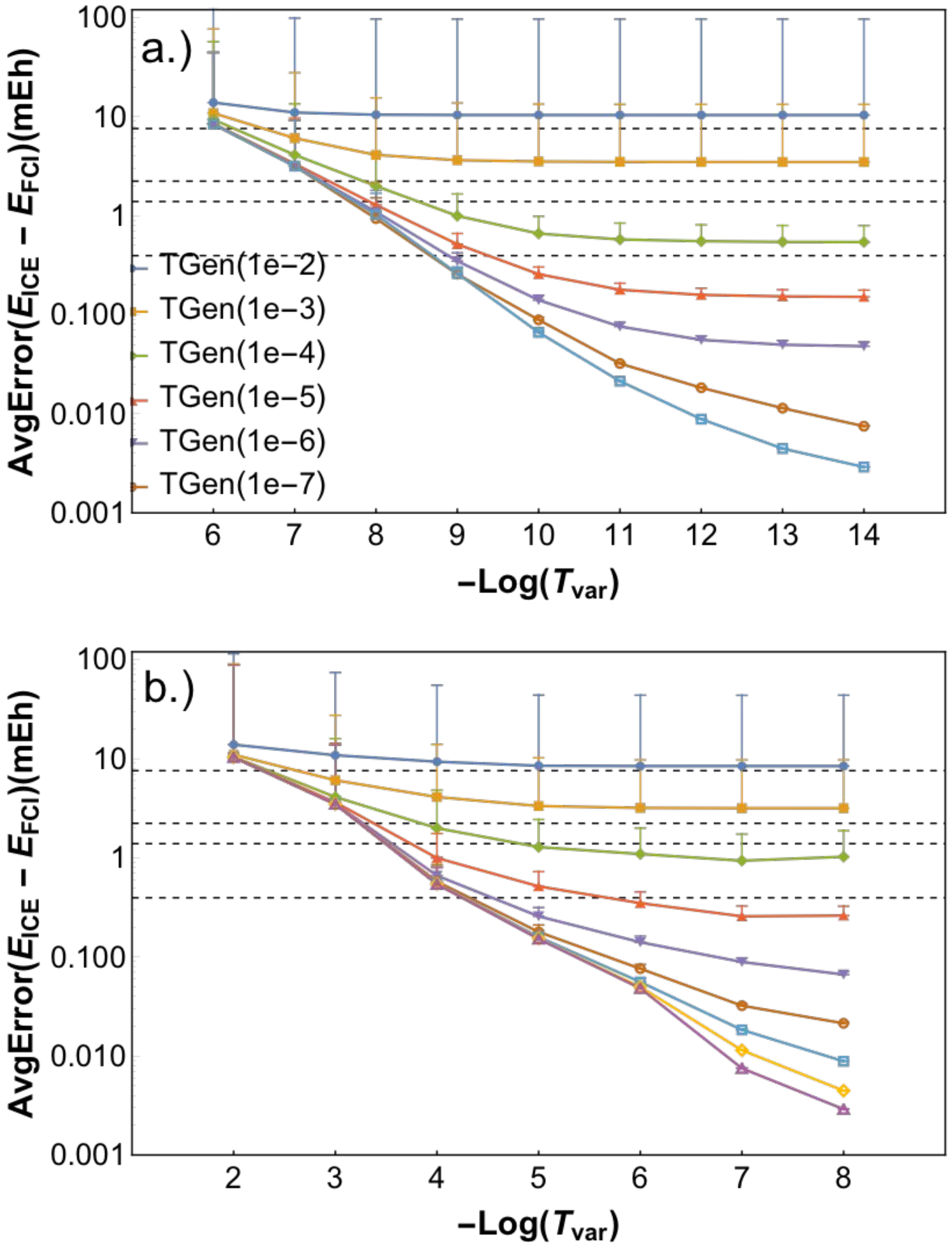

Figure 1. The mean error in energy for the DET-ICE with respect to the FCI energies for the FCI21 set. The vertical lines represent the variance in the error for the 21 molecules. Figure

a.) plots TGen vs Error with decreasing TVar and Figure b.) plots the same data but with TVar on the x-axis and decreasing TGen.

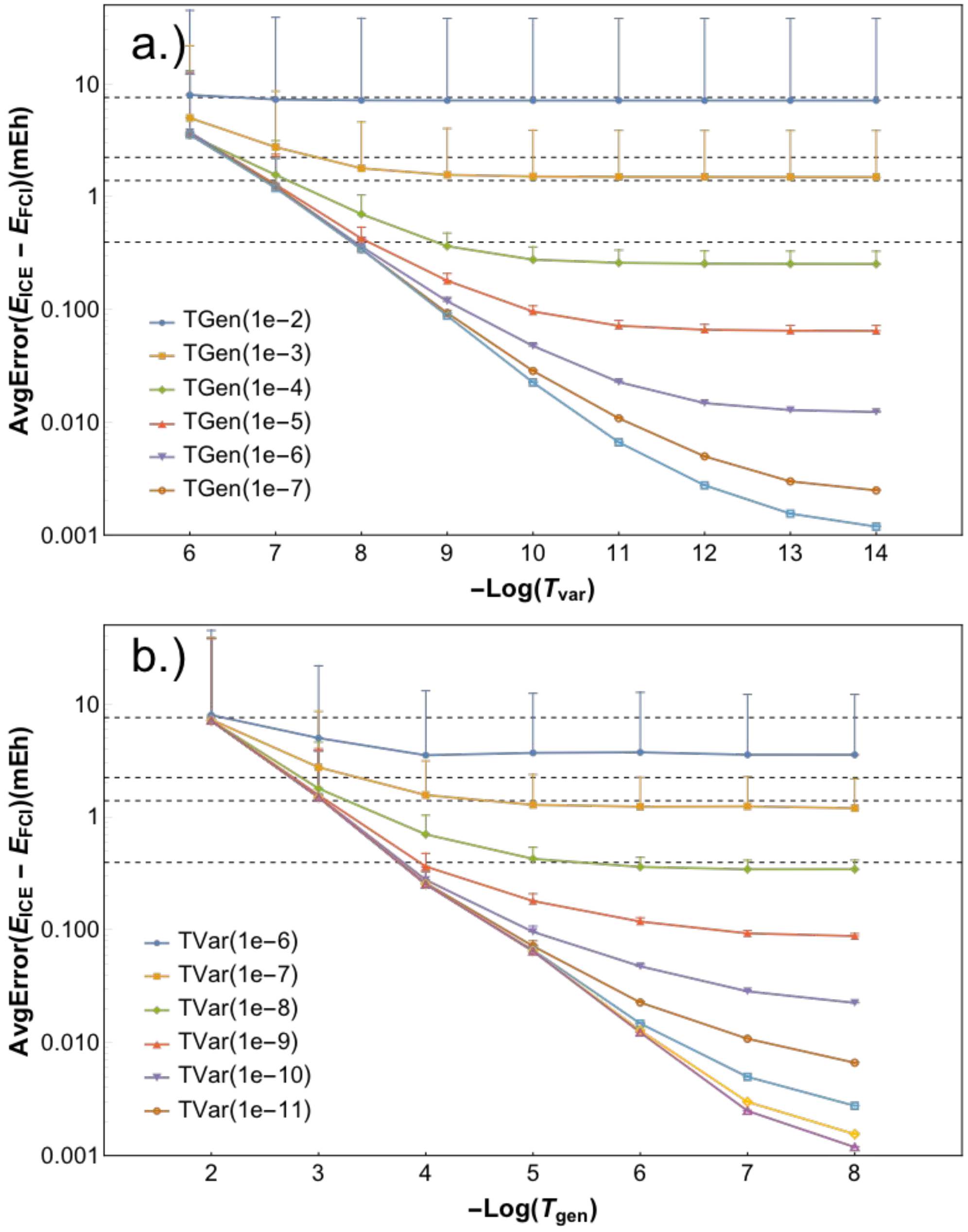

Figure 2. The mean error in energy for the CFG-ICE with respect to the FCI energies for the FCI21 set. The vertical lines represent the variance in the error for the 21 molecules. Figure a.) plots TGen vs Error with decreasing TVar and Figure b.)

plots the same data but with TVar on the x-axis and decreasing TGen.

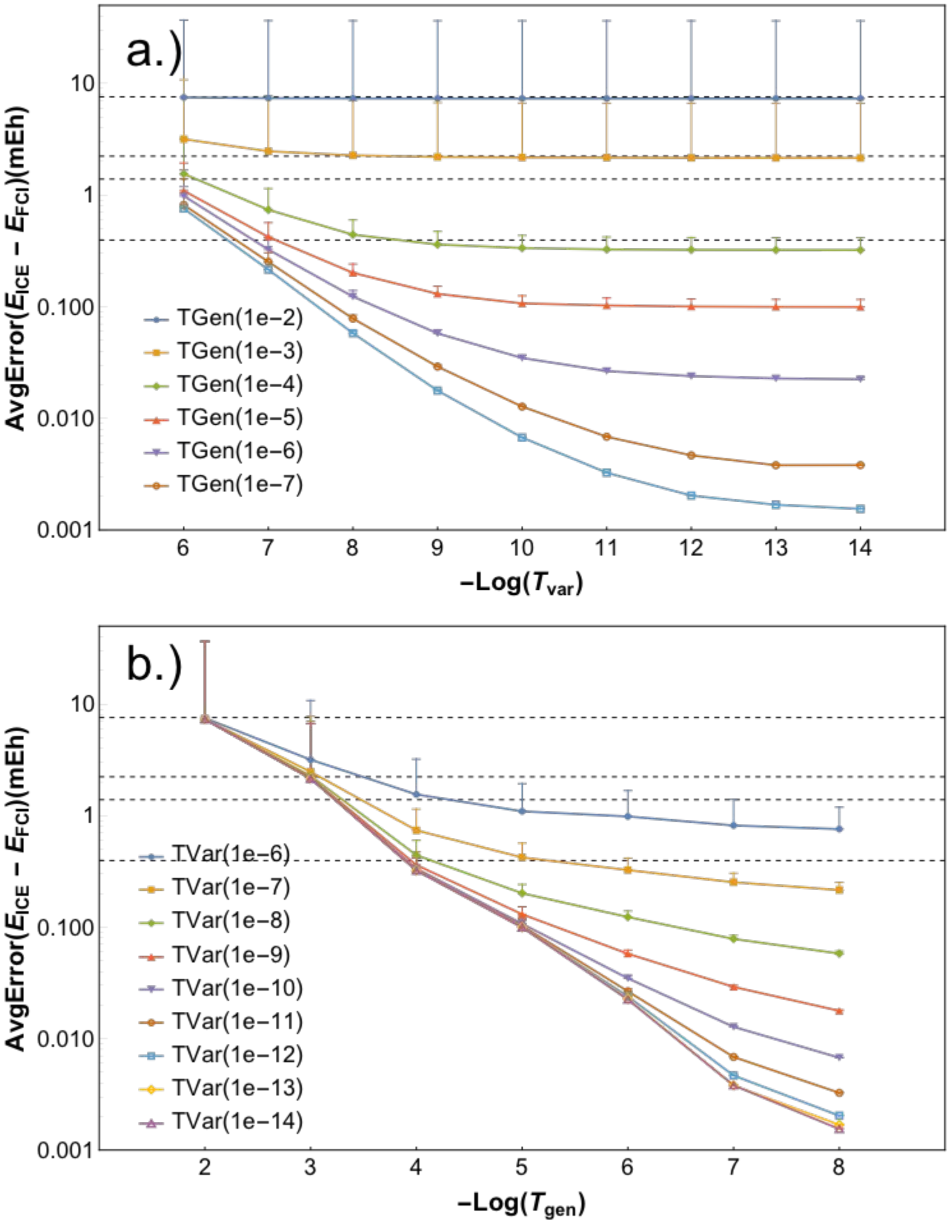

Figure 3. The mean error in energy for the CSF-ICE with respect to the FCI energies for the FCI21 set. The vertical lines

represent the variance in the error for the 21 molecules. Figure a.) plots TGen vs Error with decreasing TVar and Figure b.) plots the same data but with TVar on the x-axis and decreasing TGen.

1.2 Comparison of variational +PT2 ICE vs FCI energy

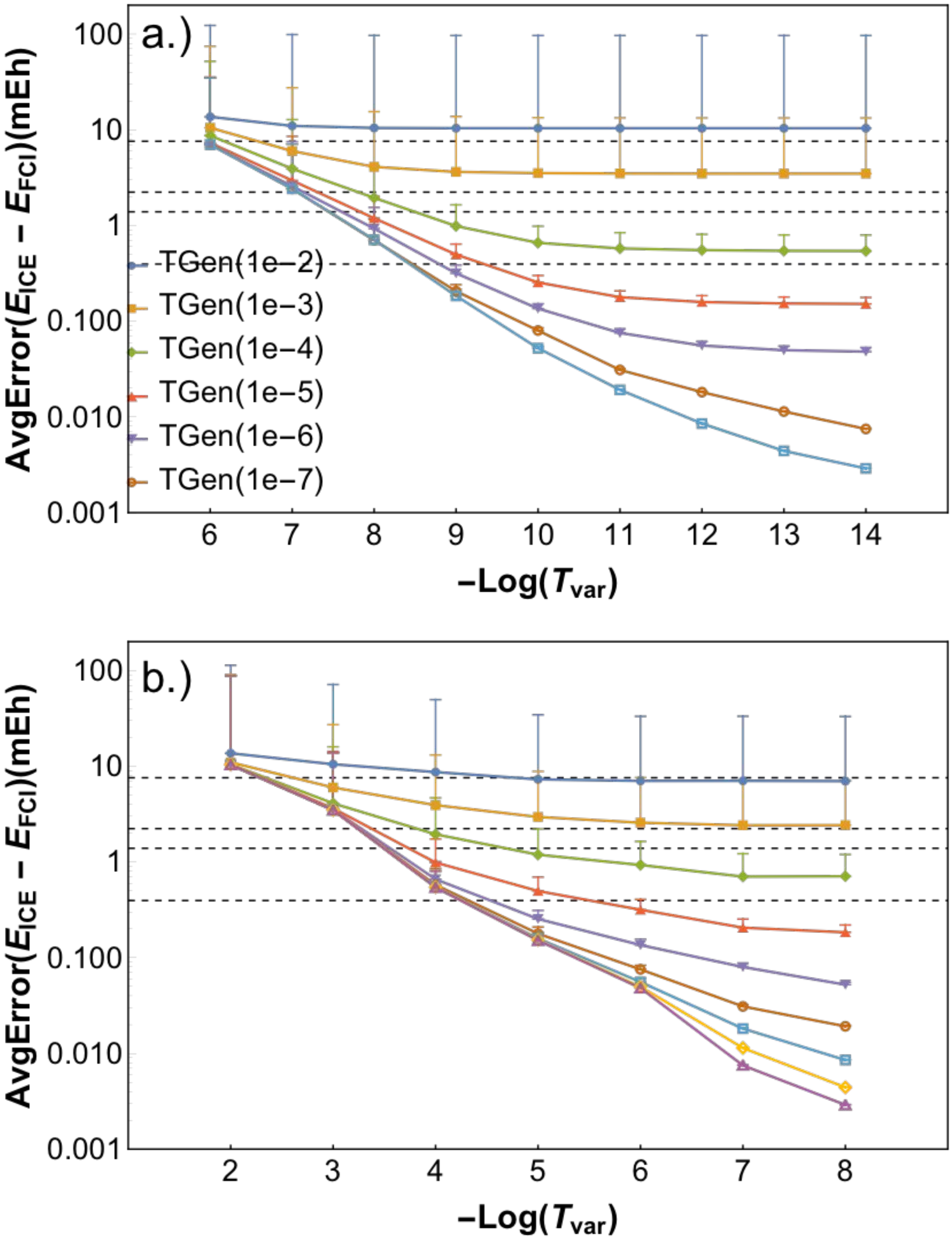

Figure 4. Absolute errors for the DET-ICE variant including the perturbative correction due to the MPBFs not included in the variational space. The figure a) gives the errors versus the

variational threshold whereas figure b) gives the error with respect to the generator thresholds.

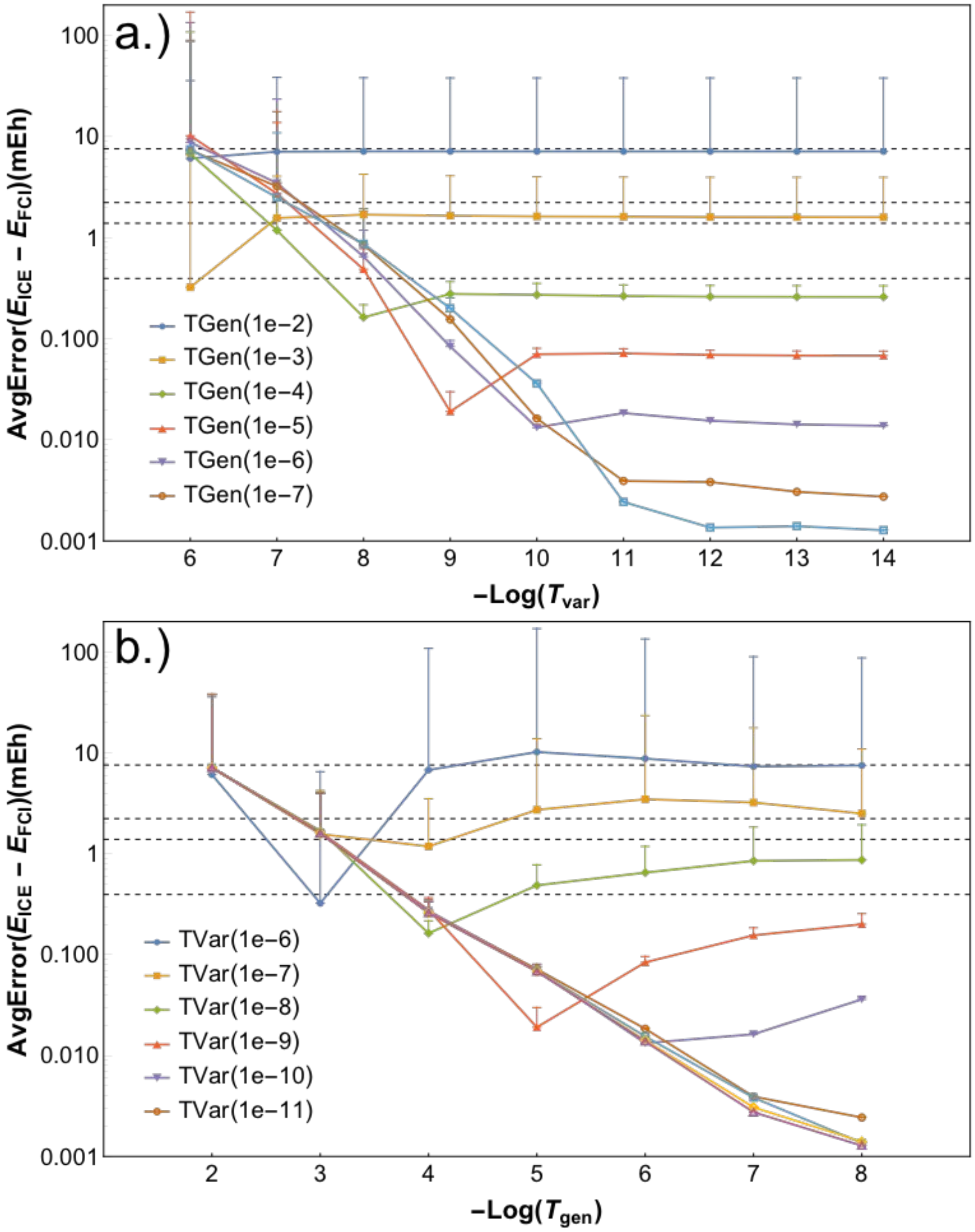

Figure 5. Absolute errors for the CFG-ICE variant including the perturbative correction due to the MPBFs not included in the

variational space. The figure a) gives the errors versus the variational threshold whereas figure b) gives the error with respect to the generator thresholds.

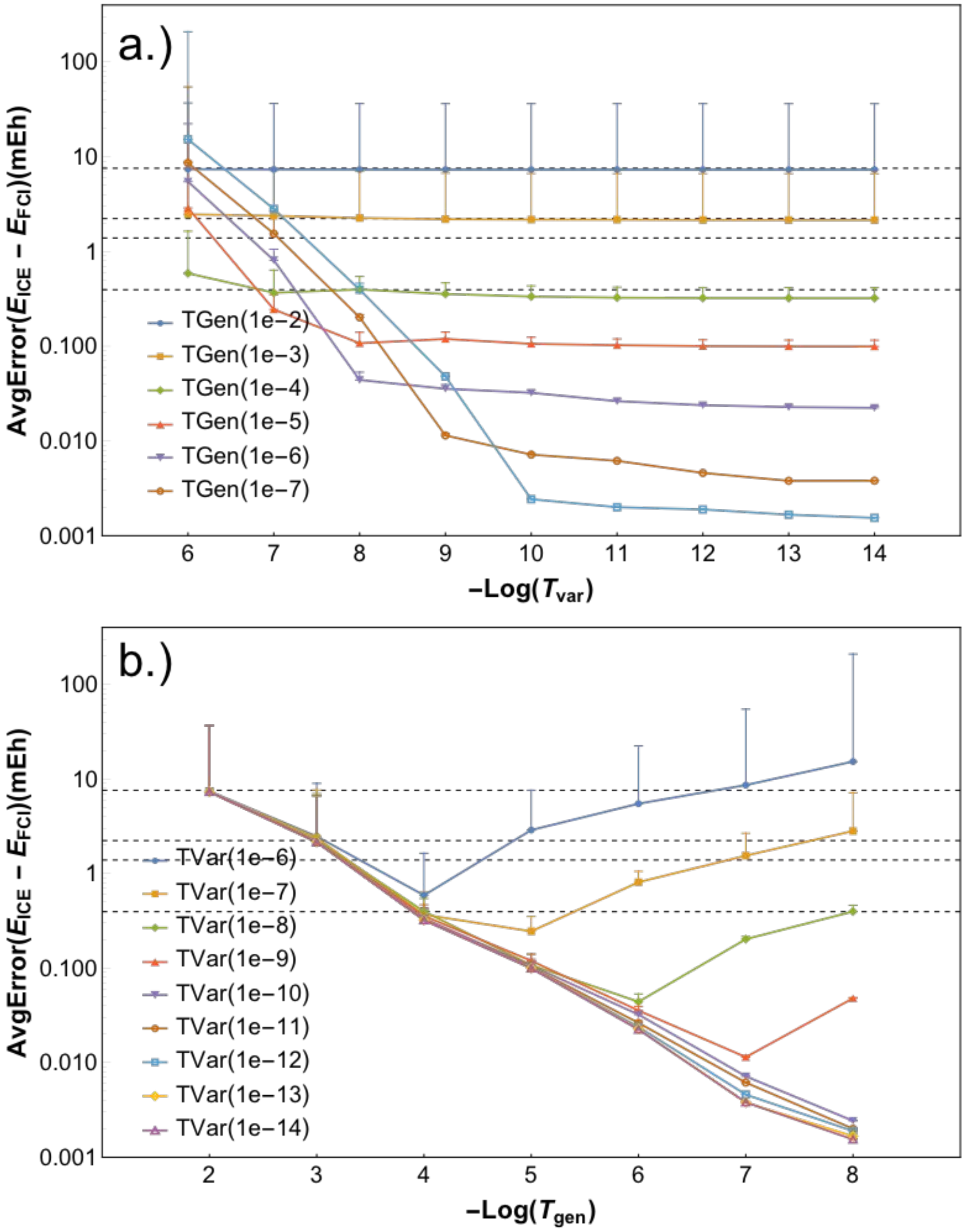

Figure 6. Absolute errors for the CSF-ICE variant including the perturbative correction due to the MPBFs not included in the variational space. The figure a) gives the errors versus the

variational threshold whereas figure b) gives the error with respect to the generator thresholds.

1.3 Comparison of variational parameters

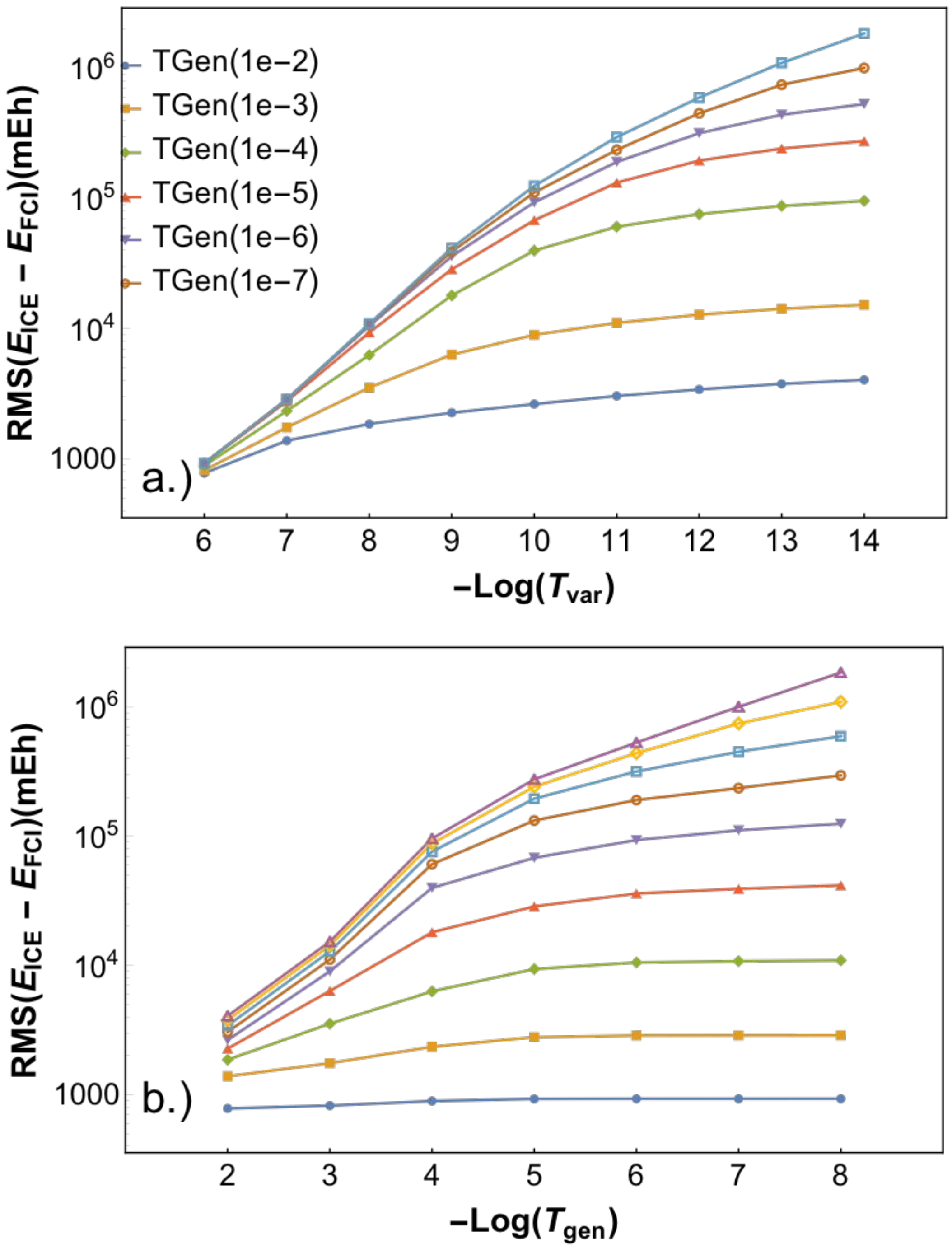

Figure 7. A comparison of total number of DETs in the variational space for the DET-ICE variant with TGen and TVar. Figure a) shows the number of DETs vs Log(TVar) and figure b) shows the number of DETs vs Log(TGen) in order to illustrate the

dependence of the number of variational parameters on TGen and TVar.

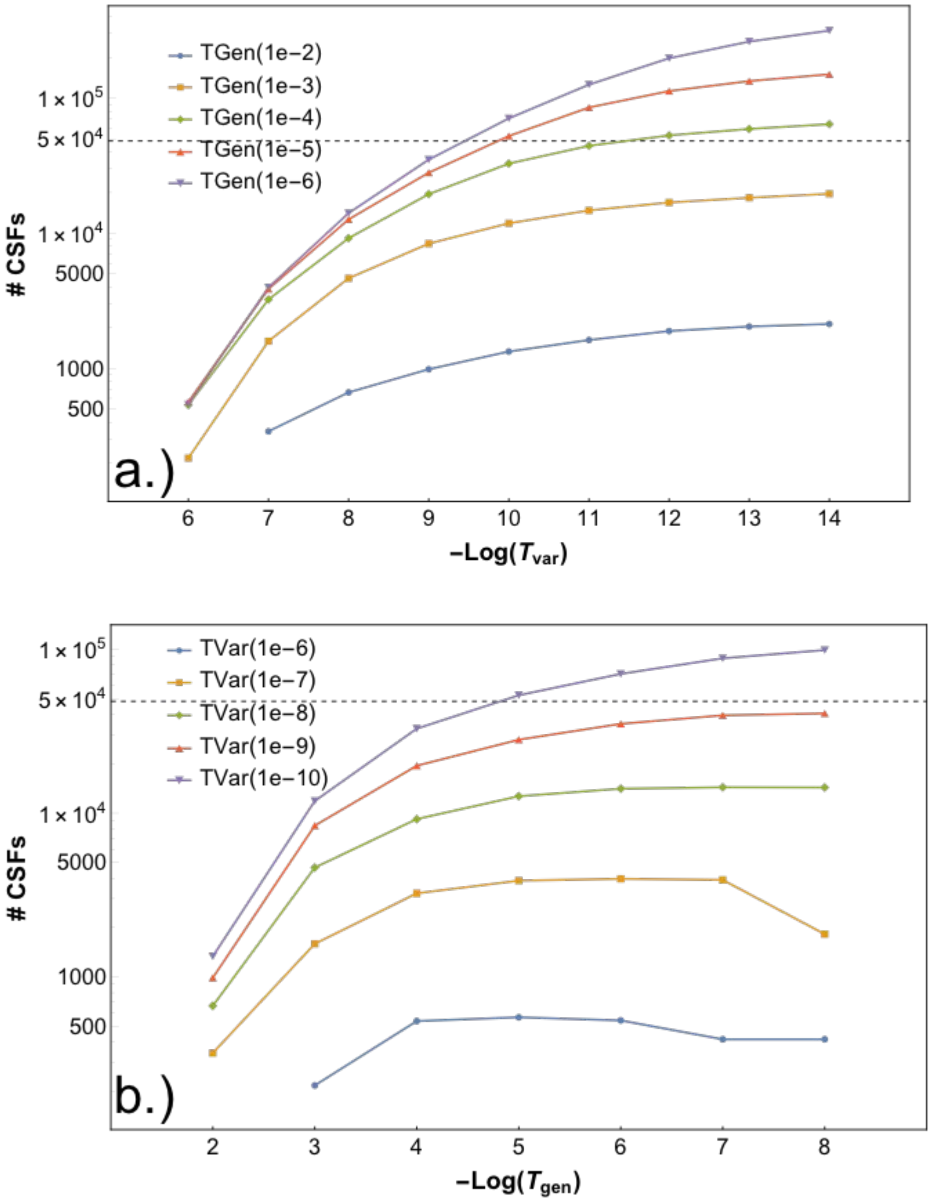

Figure 8. A comparison of total number of CSFs in the variational space for the CFG-ICE variant with TGen and TVar. Figure a) shows the number of CSFs vs Log(TVar) and figure b) shows the number of CSFs vs Log(TGen) in order to illustrate the

dependence of the number of variational parameters on TGen and TVar.

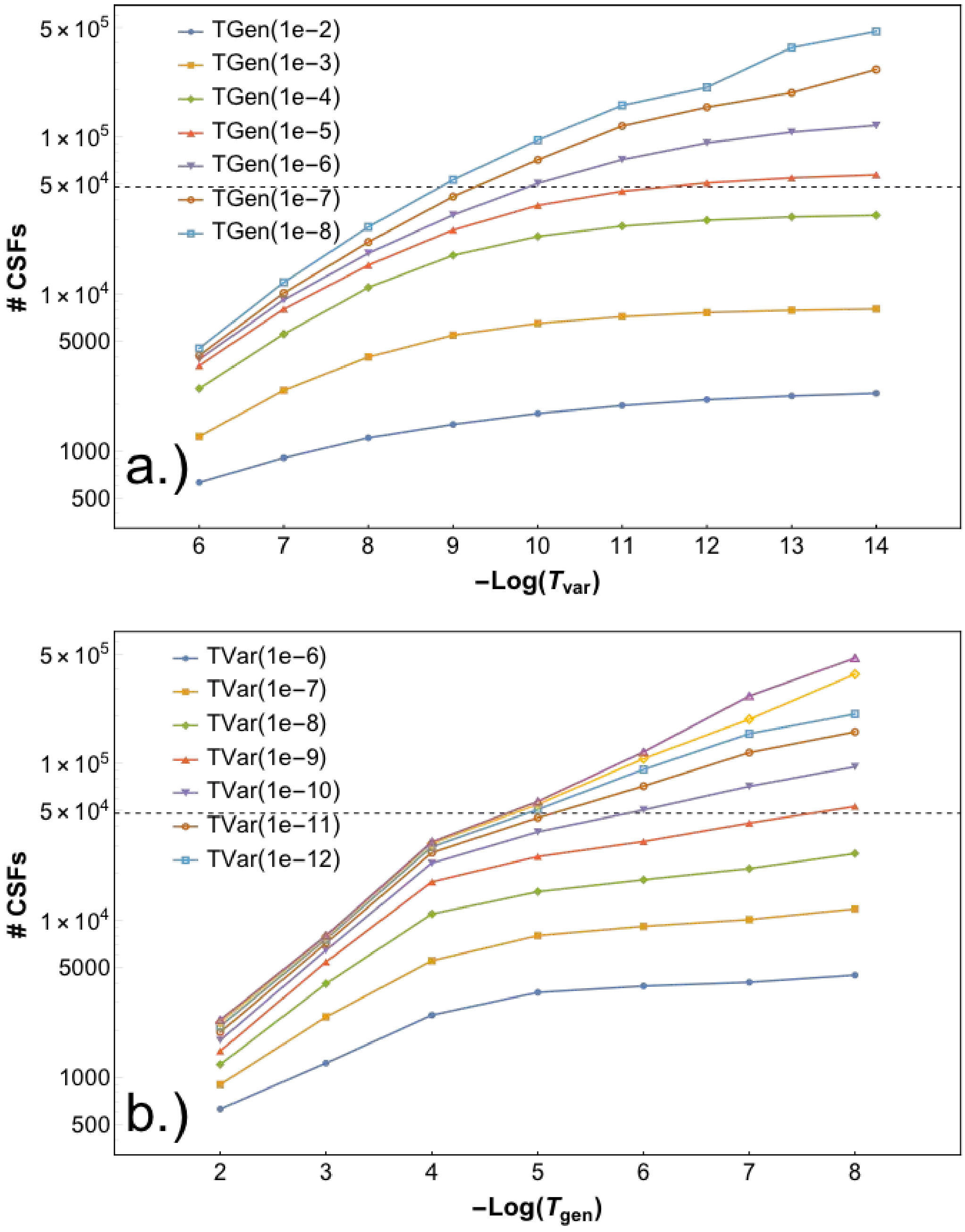

Figure 9. A comparison of total number of CSFs in the variational space for the CSF-ICE variant with TGen and TVar.

Figure a) shows the number of CSFs vs Log(TVar) and figure b) shows the number of CSFs vs Log(TGen) in order to illustrate the dependence of the number of variational parameters on TGen and TVar.

1.4 Extrapolation Scheme

1.4.1 Geometries

1.4.1.1 Methane

```
  C          0.00025019268395                    0.00013310450584
0.00011769952083
  H          0.63610258964094                    0.63612278926865
0.63610233254811
  H          -0.63608578139881                   -0.63592282199850
0.63569398574309
  H       0.63583518811787          -0.63606387588664           -
0.63601932587059
  H       -0.63610218904394           0.63573080411065          -
0.63589469194145
```

1.4.1.2 Ethene

```
 C      0.000000     0.000000     0.000000
 C      0.000000     0.000000     1.339000
 H      0.941370     0.000000     1.882500
 H     -0.941370     0.000000    -0.543500
 H      0.941370     0.000000    -0.543500
 H     -0.941370     0.000000     1.882500
```

1.4.1.3 Butadiene

H	0.000000	0.000000	0.000000
C	0.000000	0.000000	1.089000
C	1.156144	0.000000	1.756500
H	-0.943102	0.000000	1.633500
H	1.156144	0.000000	2.845500
C	2.411881	0.000000	1.031500
C	3.568025	0.000000	1.699000
H	2.411881	0.000000	-0.057500
H	4.511126	0.000000	1.154500
H	3.568025	0.000000	2.788000

1.4.2 Convergence with TGen

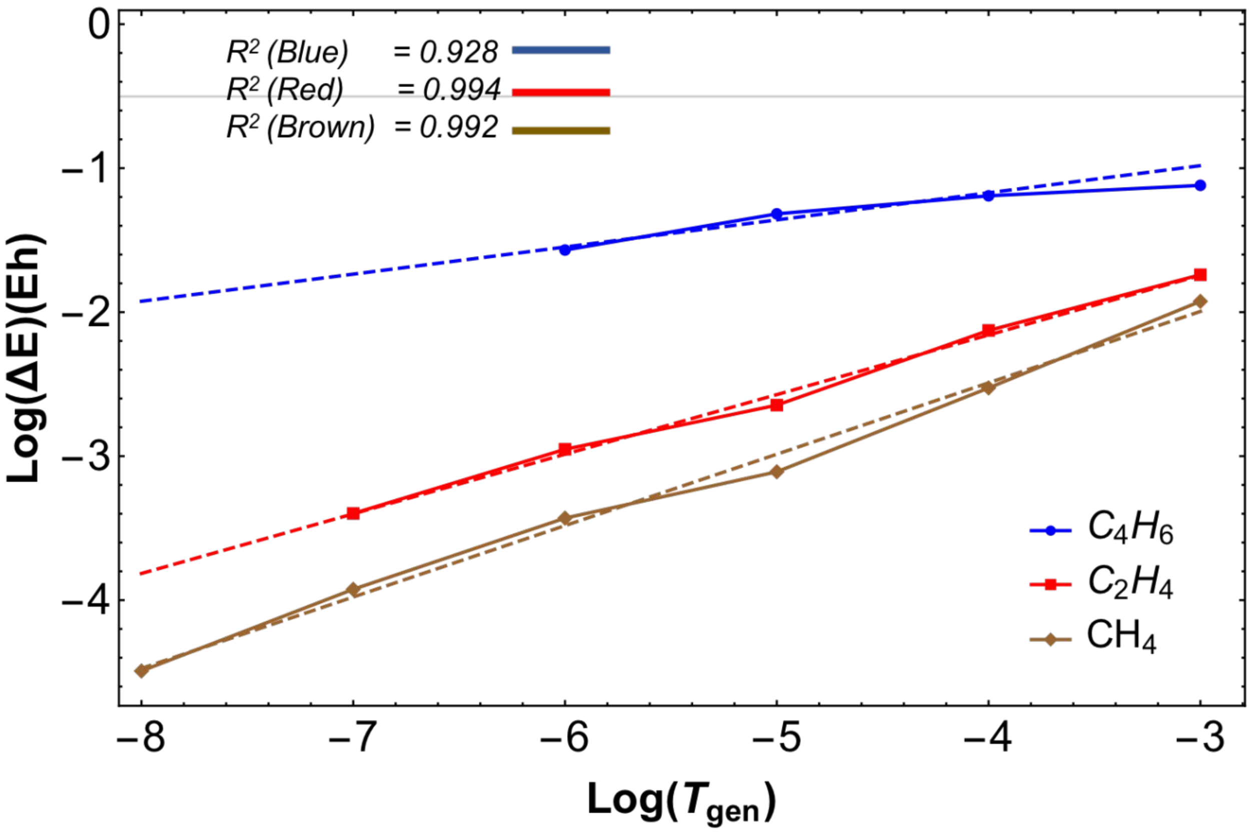

Figure 10. Fit of the errors to extrapolated FCI energies using the variational ICE energy with decreasing TGen and a fixed ratio of TVar/TGen = 10^{-3}. The figure illustrates the quality of the linear fit with R^2 = 93%, 99%, 99% for C_4H_6, C_2H_4 and CH_4 respectively.

The convergence can be seen much clearly while plotting the absolute energies vs TGen values (with TVar/TGen fixed to 10^{-3}) as shown in Figure 10.

1.4.3 Comparison of the extrapolation Scheme

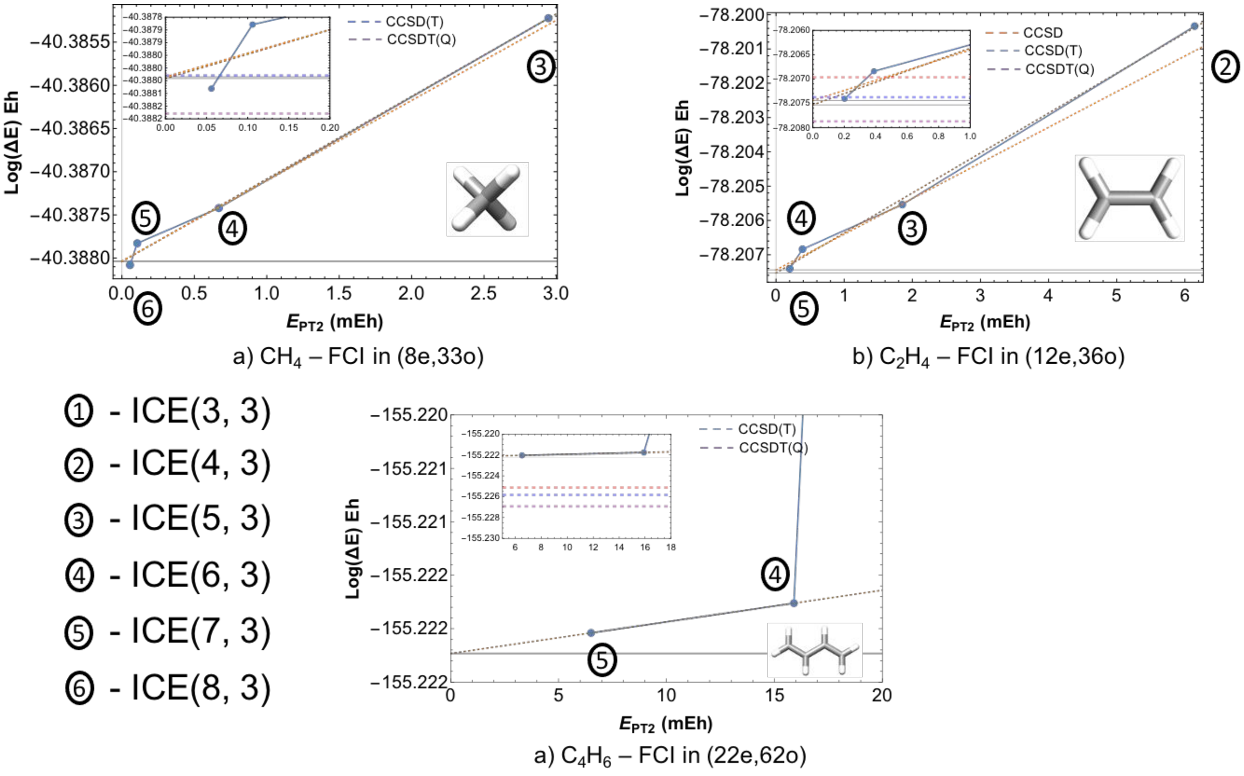

Figure 11. The extrapolated energy for the three conjugated polyene molecules (CH_4, C_2H_4 and C_4H_6) along with the coupled cluster energies. The cc-pVDZ basis is used for hydrogen atoms and SV basis for carbon atoms. The correlated number of electrons and orbitals for each system is shown also given. The ICE protocol used corresponds to ICE(TGen,τ) with $\tau = 3$ and decreasing TGen which is marked with numbered circles for each point.

1.5 Size-Inconsistency Error

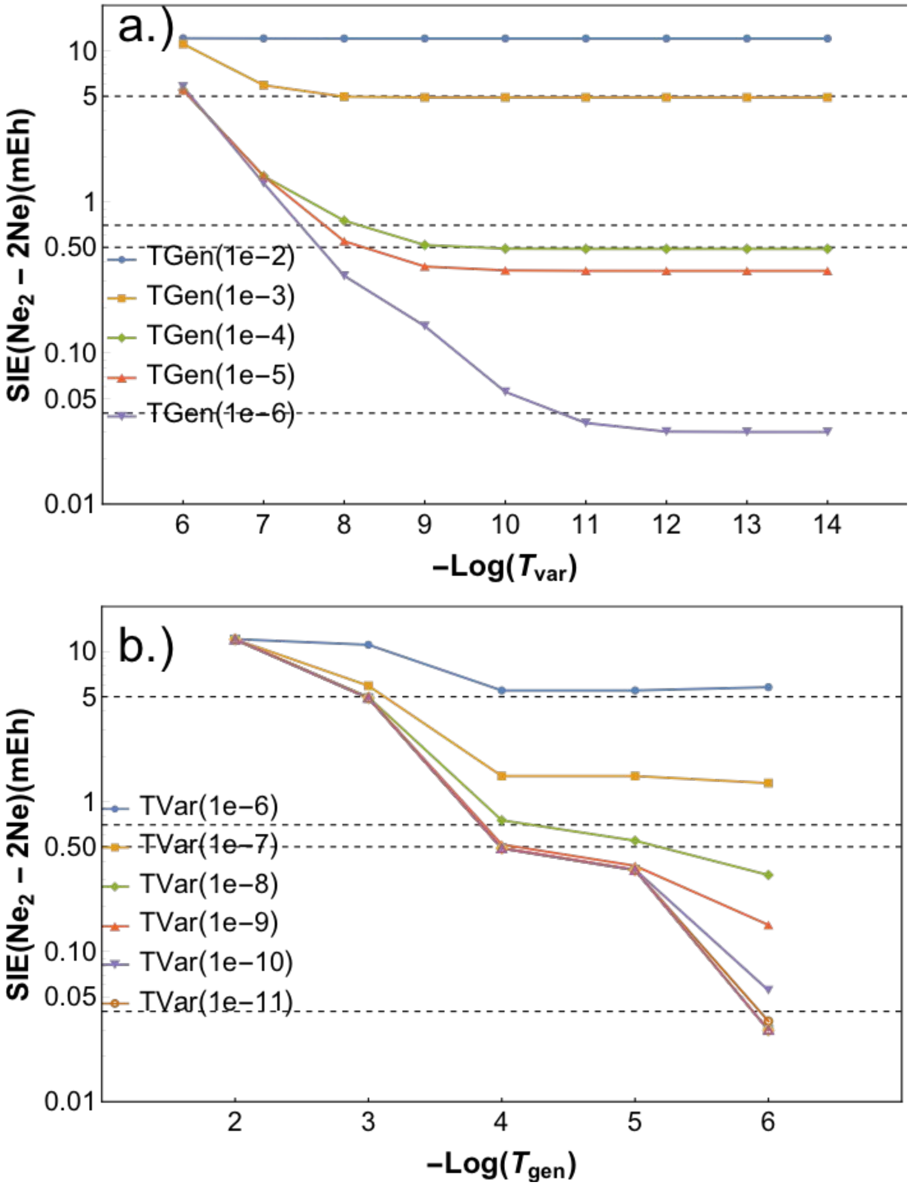

Figure 12. Size-inconsistency error (SIE) for DET-ICE variant calculated by taking the difference of Ne_2 and 2 Ne energies.

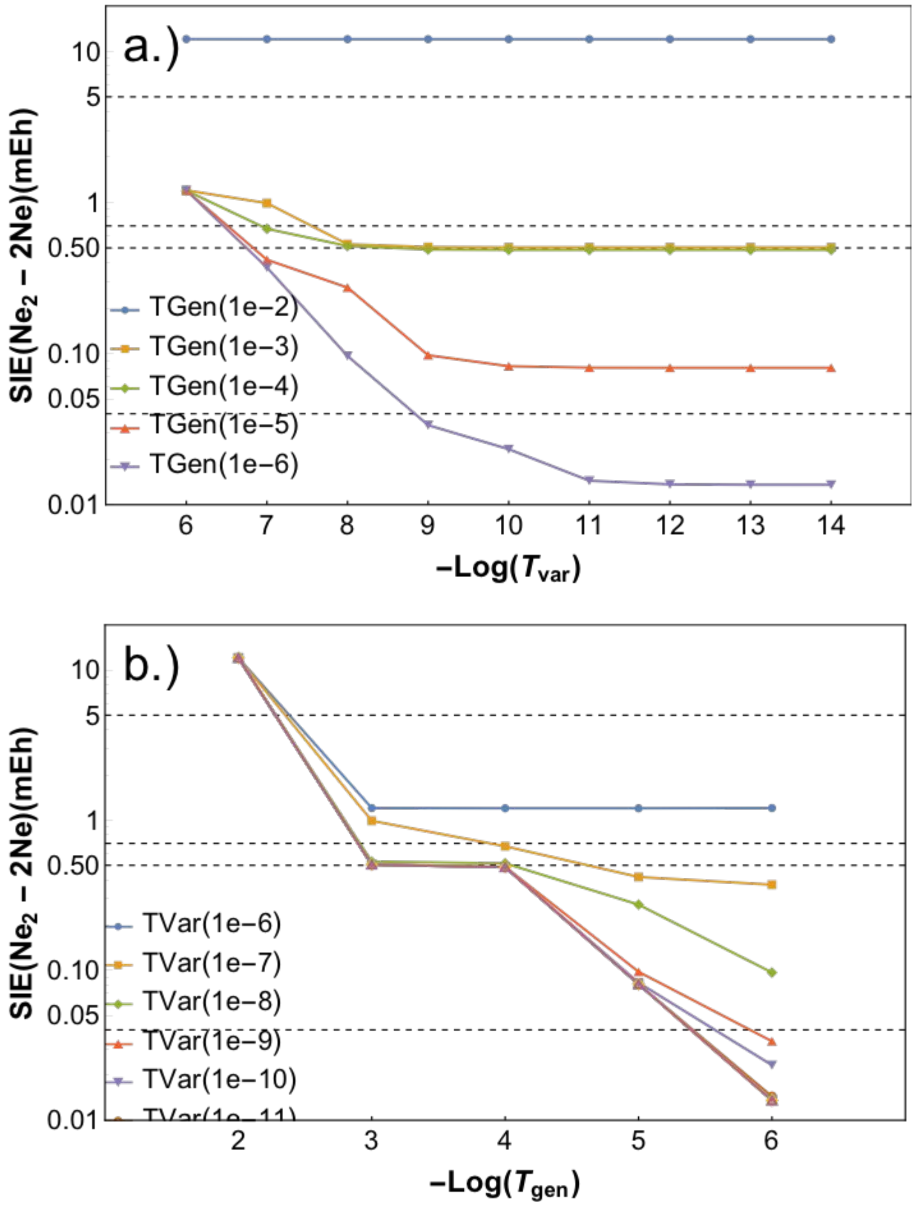

Figure 13. Size-inconsistency error (SIE) for CFG-ICE variant calculated by taking the difference of Ne_2 and 2 Ne energies.

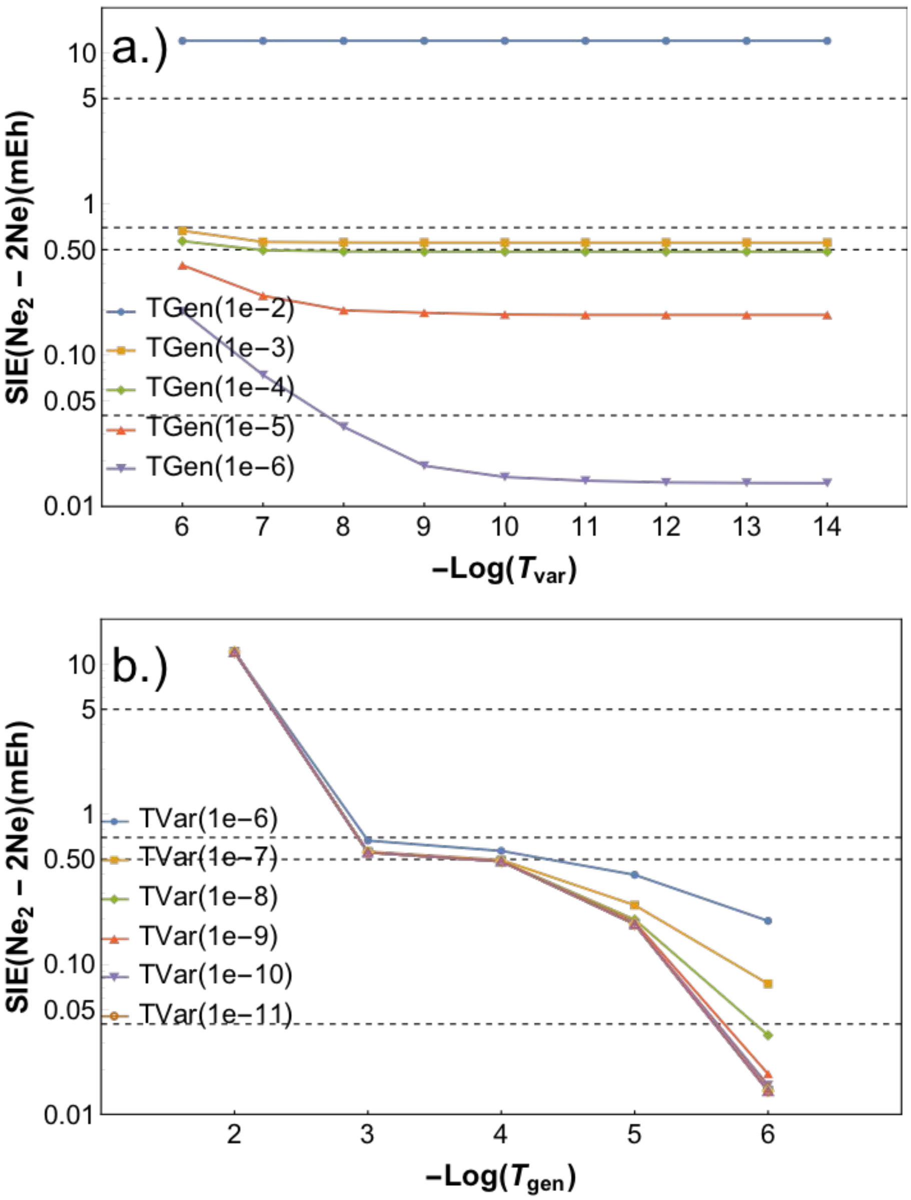

Figure 14. Size-inconsistency error (SIE) for CSF-ICE variant calculated by taking the difference of Ne_2 and 2 Ne energies.
